# SUMOylation is required for fungal development and pathogenicity in the rice blast fungus *Magnaporthe oryzae*

**DOI:** 10.1111/mpp.12687

**Published:** 2018-07-17

**Authors:** You‐Jin Lim, Ki‐Tae Kim, Yong‐Hwan Lee

**Affiliations:** ^1^ Department of Agricultural Biotechnology Seoul National University Seoul 08826 South Korea; ^2^ Center for Fungal Genetic Resources, Plant Genomics and Breeding Institute, and Research Institute of Agriculture and Life Sciences, Seoul National University Seoul 08826 South Korea

**Keywords:** *Magnaporthe oryzae*, pathogenicity, post‐translational modification, rice blast fungus, SUMOylation, ubiquitination

## Abstract

Amongst the various post‐translational modifications (PTMs), SUMOylation is a conserved process of attachment of a small ubiquitin‐related modifier (SUMO) to a protein substrate in eukaryotes. This process regulates many important biological mechanisms, including transcriptional regulation, protein stabilization, cell cycle, DNA repair and pathogenesis. However, the functional role of SUMOylation is not well understood in plant‐pathogenic fungi, including the model fungal pathogen *Magnaporthe oryzae*. In this study, we elucidated the roles of four SUMOylation‐associated genes that encode one SUMO protein (MoSMT3), two E1 enzymes (MoAOS1 and MoUBA2) and one E2 enzyme (MoUBC9) in fungal development and pathogenicity. Western blot assays showed that SUMO modification was abolished in all deletion mutants. MoAOS1 and MoUBA2 were mainly localized in the nucleus, whereas MoSMT3 and MoUBC9 were localized in both the nucleus and cytoplasm. However, the four SUMOylation‐associated proteins were predominantly localized in the nucleus under oxidative stress conditions. Deletion mutants for each of the four genes were viable, but showed significant defects in mycelial growth, conidiation, septum formation, conidial germination, appressorium formation and pathogenicity. Several proteins responsible for conidiation were predicted to be SUMOylated, suggesting that conidiation is controlled at the post‐translational level by SUMOylation. In addition to infection‐related development, SUMOylation also played important roles in resistance to nutrient starvation, DNA damage and oxidative stresses. Therefore, SUMOylation is required for infection‐related fungal development, stress responses and pathogenicity in *M. oryzae*. This study provides new insights into the role of SUMOylation in the molecular mechanisms of pathogenesis of the rice blast fungus and other plant pathogens.

## Introduction

Post‐translational modifications (PTMs) regulate diverse biological processes by the attachment of specific proteins or functional groups to various substrates (Gill, 2004). PTMs include diverse protein‐modifying processes that are widely conserved in eukaryotic organisms (Watts, 2013). In fungal plant pathogens, the most comprehensively studied PTM is phosphorylation, which is essential for appressorium formation (Dean, 1997; Flaishman *et al*., 1995; Kang *et al*., 1999, Zhao *et al*., 2007). Methylation in fungi has also been studied as a post‐translational epigenetic modification (Jeon *et al*., 2015; Martienssen and Colot, 2001). Ubiquitination is a major mechanism of protein degradation in eukaryotic organisms (Liu and Xue, 2011) and has been actively studied in various fungi, including plant pathogens (Guo *et al*., 2015; Oh *et al*., 2012; Prakash *et al*., 2016). In contrast, in‐depth studies of SUMOylation, which has components different from those of ubiquitination, are limited to the model fungus *Saccharomyces cerevisiae* and human fungal pathogens (Denison *et al*., 2005; Leach *et al*., 2011; Park *et al*., 2011).

SUMOylation involves the attachment of small ubiquitin‐related modifiers (SUMOs) to substrate proteins. This process has several important biological roles, including transcriptional regulation, protein localization and stability, cell cycle, DNA repair and stress resistance (Alonso *et al*., 2015; Johnson and Blobel, 1999; Lomeli and Vazquez, 2011). The main components of SUMOylation, other than SUMO, are SUMO‐activating enzymes (E1), SUMO‐conjugating enzymes (E2), SUMO ligases (E3) and SUMO proteases. In the SUMOylation process, the SUMO precursor is cleaved by SUMO protease (hydrolase) to reveal the diglycine motif at its C‐terminal end and to become a mature form (Hickey *et al*., 2012). SUMO is then activated by the formation of a thioester bond between a cysteine residue of E1 and a glycine residue of SUMO. SUMO is transferred from E1 to a cysteine residue of E2 by a thioester bond (Desterro *et al*., 1999; Johnson and Blobel, 1997). E3 facilitates the formation of an isopeptide bond between a glycine residue of SUMO and lysine residues of the consensus motif ΨKXE/D (Ψ and X represent a hydrophobic amino acid and any amino acid, respectively) or a non‐consensus motif on the substrate (Tatham *et al*., 2001). This isopeptide bond is cleaved by a SUMO protease (isopeptidase) (Hickey *et al*., 2012). SUMO is then recycled for use in SUMOylation (Dohmen, 2004; Lomeli and Vazquez, 2011; Sriramachandran and Dohmen, 2014). This process is similar to ubiquitination, but E1 acts as a heterodimer and E3 is not essential for the attachment of SUMO to substrates (Gill, 2004; Müller *et al*., 2001; Saracco *et al*., 2007).

SUMOylation regulates the inflammatory response in mammals, flowering time and resistance to abiotic stress in plants, and sporulation and virulence in fungi (Dohmen, 2004; Gujjula *et al*., 2016; Harting *et al*., 2013; Leach *et al*., 2011; Raorane *et al*., 2013; Wong *et al*., 2008). In *S. cerevisiae*, SMT3 plays a role as SUMO, AOS1 and UBA2 as E1 SUMO‐activating enzymes, UBC9 as an E2 SUMO‐conjugating enzyme, SIZ1, SIZ2, CST9 and MMS21 as E3 SUMO ligases, and ULP1, ULP2 and WSS1 as SUMO proteases (Mullen *et al*., 2010; Pasupala *et al*., 2012). Amongst the SUMOylation components, SMT3, AOS1, UBA2, UBC9, MMS21 and ULP1 are essential for viability (Johnson and Blobel, 1999). However, in other fungal species, such as *Schizosaccharomyces pombe*, *Aspergillus nidulans*, *A*  *flavus* and *Candida albicans*, SUMO is not essential for viability (Leach *et al*., 2011; Nie *et al*., 2016; Tanaka *et al*., 1999; Wong *et al*., 2008). Despite these functional studies, the functional roles of the SUMOylation pathway in plant‐pathogenic fungi are unclear.

*Magnaporthe oryzae* is an ascomycete plant pathogen which causes rice blast disease and is associated with a considerable socioeconomic burden (Kim *et al*., 2009). *Magnaporthe oryzae* is an important model organism for the study of plant–fungal pathogen interactions because the host and fungal genomes have been sequenced and the genomes are now applicable to molecular biological studies (Dean *et al*., 2005; Goff *et al*., 2002; Valent, 1990). The disease cycle starts when a conidium (asexual spore) attaches to a hydrophobic surface of the host plant, and germinates and forms an appressorium, a specialized infection structure, from the tip of the germ tube. The appressorium penetrates the host surface by exerting a pressure of >8 MPa (Ebbole, 2007). After colonizing the host plant, secondary inoculum is produced by conidiogenesis (Goh *et al*., 2011b). The secondary inoculum is a key factor for epidemics as *M. oryzae* is a polycyclic pathogen (Kim *et al*., 2009). A better understanding of SUMOylation in the rice blast fungus will provide new insights into infection‐related fungal development.

In this study, we identified the components of SUMOylation amongst ubiquitin‐like modifiers (UBLs) by phylogenetic analysis, and determined their functions in *M. oryzae*. The SUMOylation machinery is conserved in diverse fungal phyla. Deletion of the four genes encoding SUMO, two E1 enzymes and one E2 enzyme led to pleiotropic phenotypes, including conidiation, septum formation, sensitivity to stress and pathogenicity. Further analysis indicated that infection‐related fungal development and pathogenicity were regulated by SUMOylation. This study improves our understanding of SUMOylation in the rice blast pathogen and other plant‐pathogenic fungi.

## Results

### Phylogenetic analysis of UBLs in fungi

**Figure 1:**
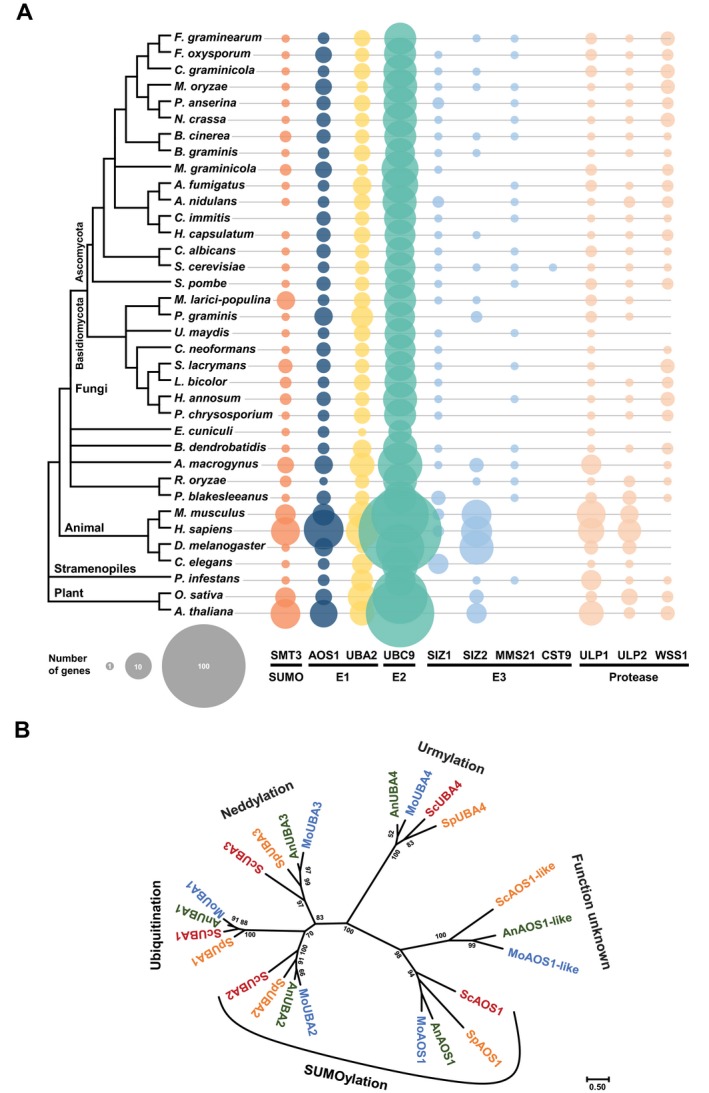
Yeast homologues of ubiquitin‐like modifiers (UBLs) in fungi. (A) A schematic species tree of the Fungal Genome Gold Standard from the Comparative Fungal Genomics Platform (CFGP; http://cfgp.riceblast.snu.ac.kr) and model organisms was constructed on the basis of the National Center for Biotechnology Information (NCBI) taxonomy. The number of yeast homologues in each species is represented by the areas of the circles. (B) Unrooted E1 domain tree of *Magnaporthe oryzae* (Mo), *Aspergillus nidulans* (An), *Schizosaccharomyces pombe* (Sp) and *Saccharomyces cerevisiae* (Sc). The tree was constructed by the maximum‐likelihood method with 1000 bootstraps.

We identified *S. cerevisiae* SUMOylation homologues in fungi by protein blast and Pfam domain analysis. The domain analysis of *S. cerevisiae* showed that SMT3 possesses a SUMO domain (PF11976), AOS1 and UBC2 possess a ThiF domain (PF00899), UBC9 contains a ubiquitin‐conjugating domain (PF00179) and the E3 enzymes possess various zinc finger domains (PF02891 and PF14634) (Table S1, see Supporting Information). Proteins with these domains were identified from the selected model organisms and fungal species. This retrieved not only SUMOylation‐associated proteins, but all UBLs, including those for neddylation and urmylation (Fig. 1A and Table S2, see Supporting Information). Fungi showed relatively fewer UBLs than animals and plants. For SUMO, only one gene was widely conserved in most fungal species, but some had more than one polyphyletic gene (Fig. S1A, see Supporting Information). Unlike SUMO, E1 enzymes were divided into six clades with specific functions, annotated on the basis of *S. cerevisiae* proteins, including UBA1 for ubiquitination, UBA3 for neddylation, UBA4 for urmylation, the two SUMOylation clades (AOS1 and UBA2) and one clade with unknown function (Figs 1B and S1B). UBC9 showed the highest diversity, but its homologues were well conserved in Ascomycetes (Fig. S1C). SIZ2 homologues were identified in all classes, but SIZ1 was identified only in fungi and animals, and MMS21 only in fungi and oomycota (Fig. S1D,E). CST9 was found only in *S. cerevisiae*. CST9 homologues were also found in *Allomyces macrogynus* and *Candida albicans* by blast, but these lacked the E3 ligase zinc‐RING finger domain (PF14634). ULP1 and ULP2 were also detected in all classes, but WSS1 was absent from animals (Fig. S1F,G). Unlike the SIZ1 and SIZ2 homologues, which were polyphyletic, the fungal ULP1 and ULP2 homologues were monophyletic (Fig. S1D,F). In *M. oryzae*, the domains of all SUMOylation components showed ≥42% sequence similarity with corresponding *S. cerevisiae* components (Table S3, see Supporting Information). The homologues of *M. oryzae* SUMOylation components with the highest levels of similarity were named after the *S. cerevisiae* genes.

### SUMO activation and conjugation enzymes are essential for SUMOylation in *M. oryzae*

**Figure 2:**
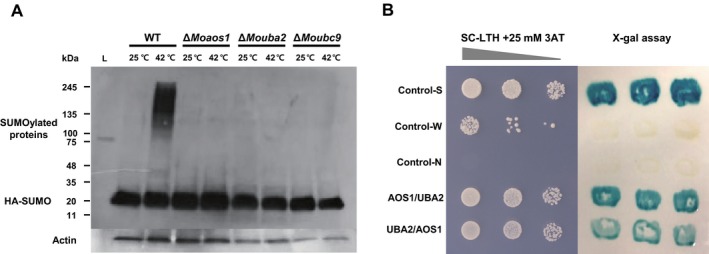
SUMOylated proteins in wild‐type (WT), Δ*Moaos1*, Δ*Mouba2* and Δ*Moubc9*, and yeast two‐hybrid (Y2H) assay of MoAOS1 and MoUBA2. (A) Protein extracts from the WT and deletion mutants were separated by sodum dodecylsulfate‐polyacrylamide gel electrophoresis (SDS‐PAGE) and subjected to Western blot analysis using an anti‐haemagglutinin (HA) antibody. (B) Y2H assay of the interaction of MoAOS1 and MoUBA2 (Control‐S, strong interaction; Control‐W, weak interaction; Control‐N, no interaction). X‐gal, 5‐Bromo‐4‐Chlroro‐3‐Indolyl‐D‐Galactopyranoside.

To confirm that the identified SUMOylation‐associated genes are important for SUMOylation in *M. oryzae*, we generated targeted deletion mutants in the genes encoding SUMO (MoSMT3), E1 (MoAOS1 and MoUBA2) and E2 (MoUBC9) by double‐joint polymerase chain reaction (PCR) and homologous recombination. The single‐gene deletion mutants were generated by inserting a hygromycin resistance cassette, and a targeted double‐gene deletion mutant was generated by inserting a geneticin resistance cassette into the single‐gene deletion mutants. Deletion of the target genes was confirmed by Southern blotting and quantitative reverse transcription‐polymerase chain reaction (qRT‐PCR) (Fig. S2, see Supporting Information). In previous studies, the levels of SUMO‐modified substrates increased on exposure to high temperature, nutrient, DNA damage and oxidative stresses (Leach *et al*., 2011; Lewicki *et al*., 2015). Therefore, thermal stress was applied to the wild‐type and to Δ*Moaos1*, Δ*Mouba2* and Δ*Moubc9*. A plasmid (pHA‐SMT3) was constructed by fusing MoSMT3 with a haemagglutinin (HA) tag and the native promoter of *MoSMT3*, and was transformed into the wild‐type and single‐gene deletion mutants. The phenotypes of the deletion mutants and the wild‐type were unaffected by transformation. This suggests that HA‐tagged MoSMT3 does not affect any other biological processes. Western blotting indicated that SUMOylation in the wild‐type was dramatically greater during mycelial growth and conidiation at high temperatures than at 25 °C (Figs 2A and S3, see Supporting Information). The level of SUMOylation was also increased under oxidative stress (100 mm H_2_O_2_) (Fig. S4, see Supporting Information). However, the levels of SUMOylated proteins in the deletion mutants were significantly decreased at high temperatures (Fig. 2A). These results indicate that MoSMT3 acts as SUMO, and that MoAOS1, MoUBA2 and MoUBC9 are indispensable for SUMOylation in *M. oryzae*. Ubiquitination‐associated components were included in the predicted SUMOylation machineries (Table S2). In contrast, deletion of MoSMT3, MoAOS1, MoUBA2 and MoUBC9 did not affect the level of ubiquitination, suggesting that they are specific to SUMOylation (Fig. S5, see Supporting Information).

Interaction of the AOS1 and UBA2 subunits of E1 heterodimers is required for SUMO activation in eukaryotic organisms, including humans, *Arabidopsis thaliana* and *S. cerevisiae* (Gill, 2004). To determine whether MoAOS1 and MoUBA2 interact as heterodimers, we performed a yeast two‐hybrid (Y2H) assay. AOS1 and UBA2 were cloned into both AD and BD vectors. After co‐transformation, MoAOS1/MoUBA2 and MoUBA2/MoAOS1 were selected on synthetic complete medium lacking leucine and tryptophan (SC‐Leu/Trp) medium and confirmed by PCR amplification. AOS1 and UBA2 strongly interacted on synthetic complete medium lacking leucine, tryptophan and histidine (SC‐Leu/Trp/His) + 25 mm 3‐amino‐1,2,4‐triazole (3AT) medium. The interactions of MoAOS1 and MoUBA2 were confirmed by 5‐Bromo‐4‐Chlroro‐3‐Indolyl‐D‐Galactopyranoside (X‐gal) assay (Fig. 2B). These results indicate that MoAOS1 and MoUBA2 are subunits of the E1 activating enzyme complex, similar to ScAOS1 and ScUBA2.

### The SUMOylation machinery is essential for fungal development

**Figure 3:**
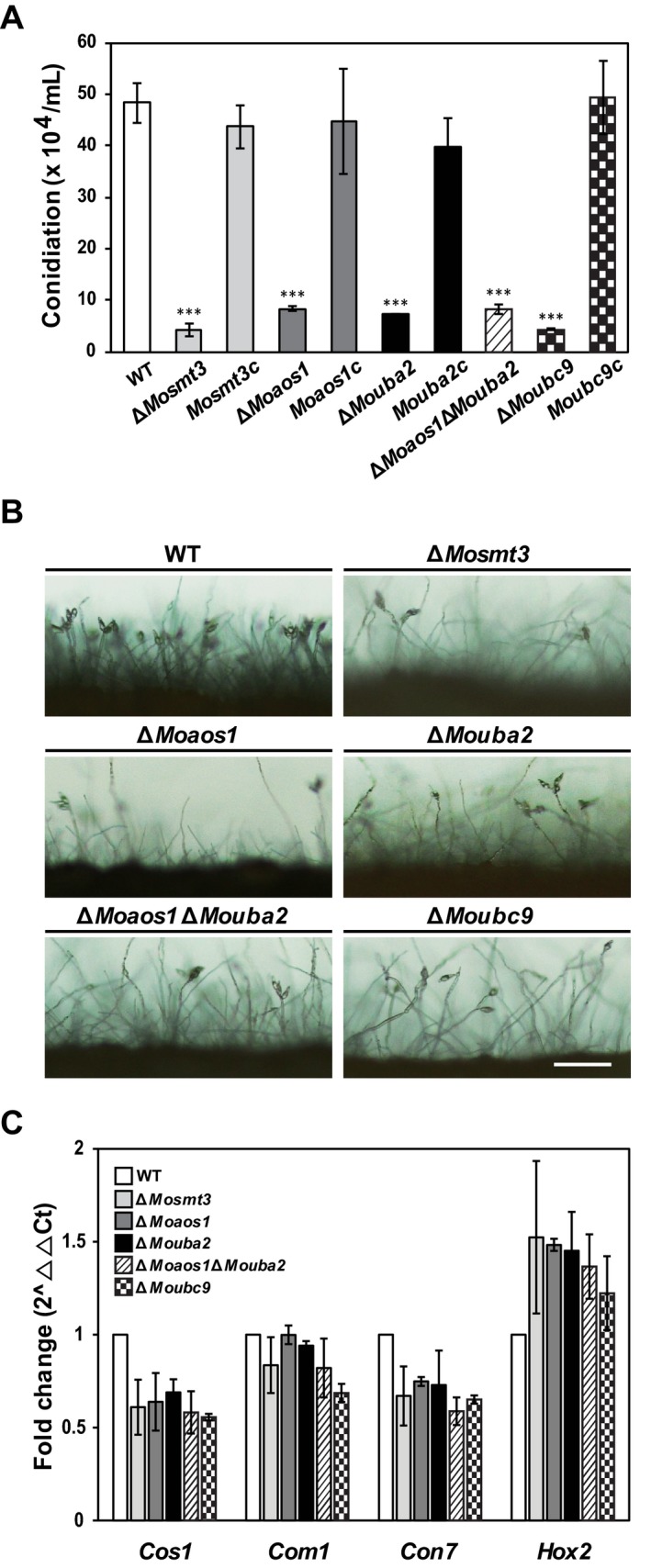
Conidiation of the wild‐type (WT), Δ*Mosmt3*, Δ*Moaos1*, Δ*Mouba2* and Δ*Moubc9* in *Magnaporthe oryzae*. (A) Conidia of the WT, deletion mutants and complemented strains were collected from V8 agar after incubation for 7 days. Significance was determined by *t*‐test (***P < 0.001). (B) Conidiogenesis on conidiophores was observed under a microscope. Scale bar, 100 µm. (C) Expression of conidiation‐related genes during conidiation was quantified by quantitative reverse transcription‐polymerase chain reaction (qRT‐PCR).

To assess the biological function of SUMOylation in *M. oryzae*, we generated five Δ*Mosmt3*, two Δ*Moaos1*, two Δ*Mouba2*, eight Δ*Moaos1*Δ*Mouba2* and three ΔMoubc9, as explained above. The phenotype of each deletion mutant was the same. We also generated complemented strains for all deletion mutants. In comparison with the wild‐type, all deletion mutants showed significant defects in mycelial growth, conidial size, conidiation, conidial germination and appressorium formation (Table S4, see Supporting Information). Mycelial growth of the deletion mutants was reduced by 10%–25% compared with the wild‐type (Table S4). The number of conidia was counted to quantitatively confirm the production of conidia. All of the deletion mutants produced (4–8) × 10^4^ conidia/mL, which was markedly lower than that of the wild‐type (4.8 × 10^5^ conidia /mL) (Fig. 3A). To identify the reason for the reduced conidial formation, we observed conidiophores under a microscope. In the wild‐type, conidia were produced in a sympodial pattern on the conidiophores, but the deletion mutants produced none, one or two conidia on the conidiophores (Fig. 3B). To further assess the defects in conidiation, the expression levels of conidiation‐related genes (*Cos1*, *Com1*, *Con7* and *Hox2*) were examined in the wild‐type and deletion mutants (Kim *et al*., 2009; Odenbach *et al*., 2007; Yang *et al*., 2010; Zhou *et al*., 2009). However, the expression levels of *Cos1*, *Com1*, *Con7* and *Hox2* did not differ between the wild‐type and deletion mutants (Fig. 3C).

**Figure 4:**
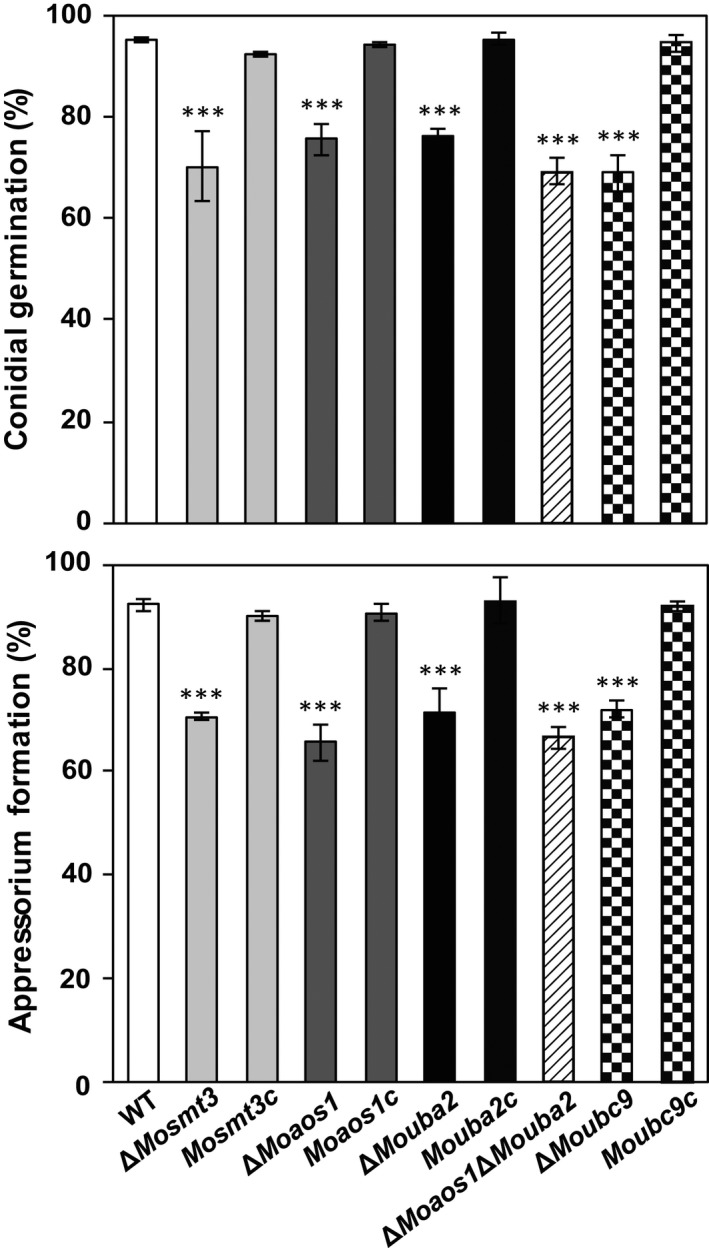
Conidial germination and appressorium formation of the wild‐type (WT), Δ*Mosmt3*, Δ*Moaos1*, Δ*Mouba2* and Δ*Moubc9* in *Magnaporthe oryzae*. Percentage of conidial germination and appressorium formation on a hydrophobic surface after incubation for 2 and 8 h, respectively. Significance was determined by *t*‐test (***P < 0.001).

In the wild‐type, 95% conidial germination and 92% appressorium formation were observed on a hydrophobic surface after 2 and 8 h of incubation, respectively. In contrast, the frequencies of conidial germination and appressorium formation were 69%–79% and 65%–71% in the deletion mutants at 2 and 8 h, respectively (Fig. 4). However, following incubation for a further 24 h, 84%–89% of the deletion mutant conidia germinated and 70%–77% of the germ tubes formed appressoria (Table S5, see Supporting Information). Thus, we performed a conidial adhesion test to assess the cause of the delayed conidial germination and appressorium formation. Compared with the wild‐type (91%), conidia of the deletion mutants (54%–76%) were ineffectively attached to the hydrophobic surface of gelbond film. These results suggest that ineffective conidial adhesion is responsible for delayed conidial germination and appressorium formation in the deletion mutants. These pleiotropic phenotypes were recovered in the complemented strains (Fig. S6, Tables S4 and S5, see Supporting Information).

### SUMOylation‐associated genes are involved in septum formation

**Figure 5:**
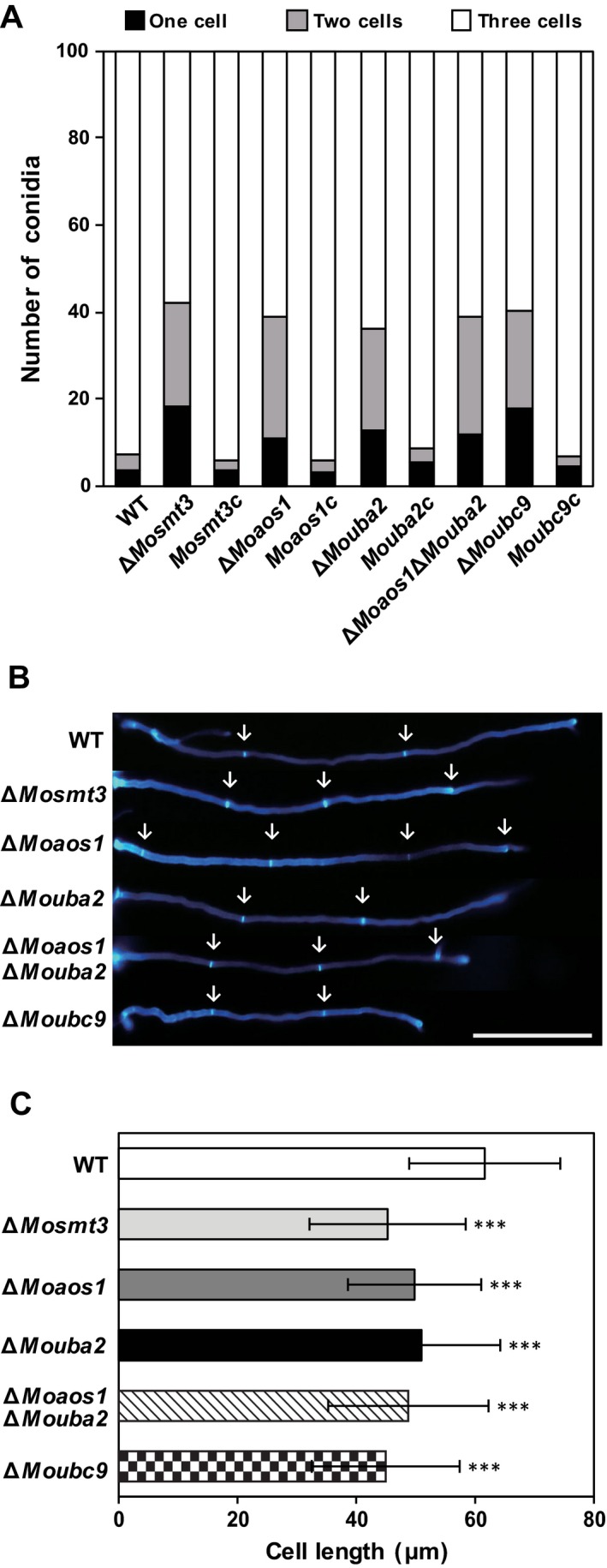
Number of cells in conidia and cell length in hyphae. (A) Percentage of conidia with one, two or three cells in the wild‐type (WT), deletion mutants and complemented strains. (B) Hyphae were stained with Calcofluor white (CFW) and observed under a fluorescence microscope. White arrows indicate septa of hyphae; scale bar, 200 μm. (C) Cell length of hyphae was measured using ImageJ. Significance was determined by *t*‐test (***P < 0.001).

To examine the roles of SUMOylation‐associated genes in septum formation in *M. oryzae*, conidia septa were observed under a microscope. In the wild‐type, most conidia (93%) had three cells, compared with 58%–64% in the deletion mutants, in which 23%–28% and 11%–18% had two and one cell, respectively (Fig. 5A). Moreover, conidia of the deletion mutants were significantly shorter than those of the wild‐type (Table S4). The abnormal conidial morphology was recovered in the complemented strains (Fig. 5A and Table S4). Hyphal growth of the deletion mutants was observed under a microscope after staining with Calcofluor white (CFW). In contrast with the wild‐type (60 μm), the cell length of hyphae between two septa was markedly shorter in the deletion mutants (45–50 μm) (Fig. 5B,C). These results suggest that SUMOylation‐associated genes are involved in septum formation of conidia and hyphae in *M. oryzae*.

### The SUMOylation components are important for stress tolerance

**Figure 6:**
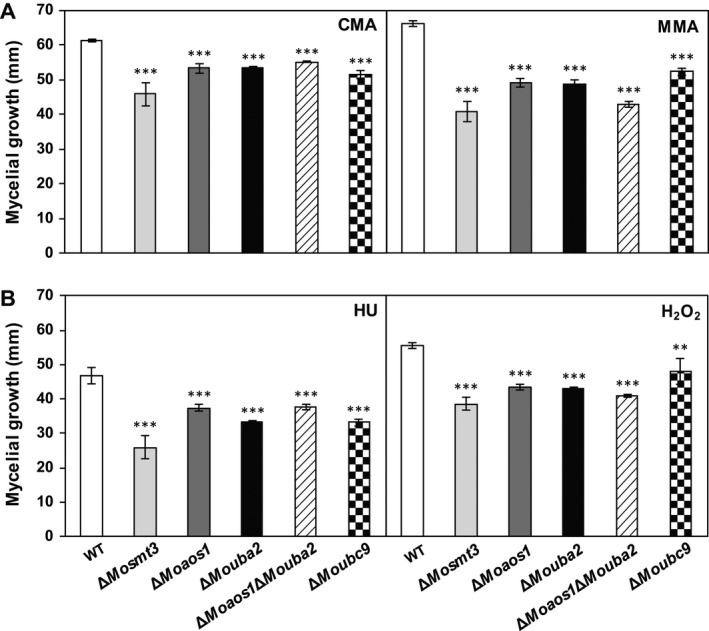
Mycelial growth under stress conditions. (A) Nutrient starvation stress on minimal agar medium (MMA) and complete agar medium (CMA). (B) DNA damage and oxidative stress on CMA containing 10 mm hydroxyurea (HU) and 5 mm H_2_O_2_. Significance was determined by *t*‐test (**P < 0.05 and ***P < 0.001). WT, wild‐type.

SUMO deletion mutants of *S. pombe* and *A. nidulans* are sensitive to heat and DNA damage stresses (Tanaka *et al*., 1999; Wong *et al*., 2008). To investigate the role of SUMOylation‐associated genes in stress resistance in *M. oryzae*, we measured mycelial growth after culture of the wild‐type and deletion mutants with the following stresses: minimal agar medium (MMA) for nutrient starvation stress, 10 mm hydroxyurea (HU) and 0.05% methyl methanesulfonate (MMS) for DNA damage stress, and 5 mm H_2_O_2_ and 3 mm methyl viologen (MV) for oxidative stress (Fig. S7, see Supporting Information). The sensitivity of Δ*Mosmt3*, Δ*Moaos1*, Δ*Mouba2* and Δ*Moaos1*Δ*Mouba2*, but not Δ*Moubc9*, to the nutrient starvation and oxidative stresses was greater than that of the wild‐type (Figs 6A, B and S7). The sensitivity of all of the deletion mutants to DNA damage stress was also higher than that of the wild‐type (Figs 6A, B and S7). These sensitivities to stress conditions were recovered in the complemented strains (Fig. S7). These results indicate that SUMOylation components are involved in stress resistance.

### SUMO, E1 and E2 are required for functional appressoria and fungal pathogenicity

**Figure 7:**
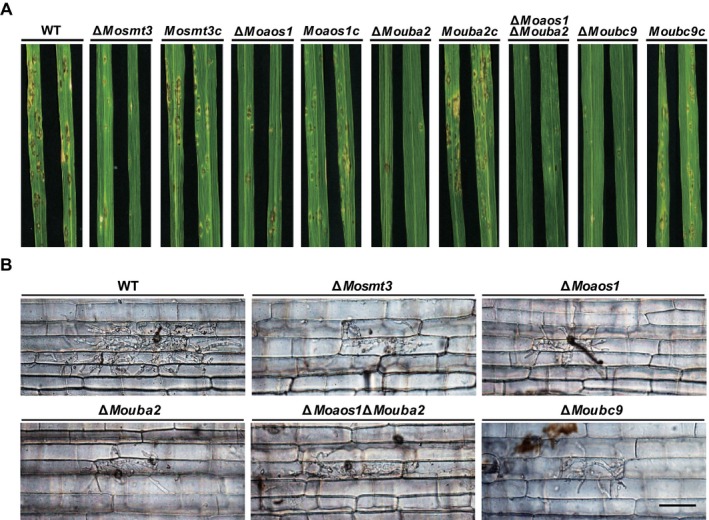
Pathogenicity assays by spray and sheath inoculation. (A) Conidial suspensions (5 × 10^4^/mL) were sprayed onto 4‐week‐old rice seedlings, and lesions were observed at 6 days post‐inoculation (dpi). (B) Conidial suspensions (2 × 10^4^/mL) were inoculated onto rice sheath cells. The growth of invasive hyphae (IH) was observed under a microscope at 48 h post‐inoculation (hpi). Scale bar, 50 μm. WT, wild‐type.

**Figure 8:**
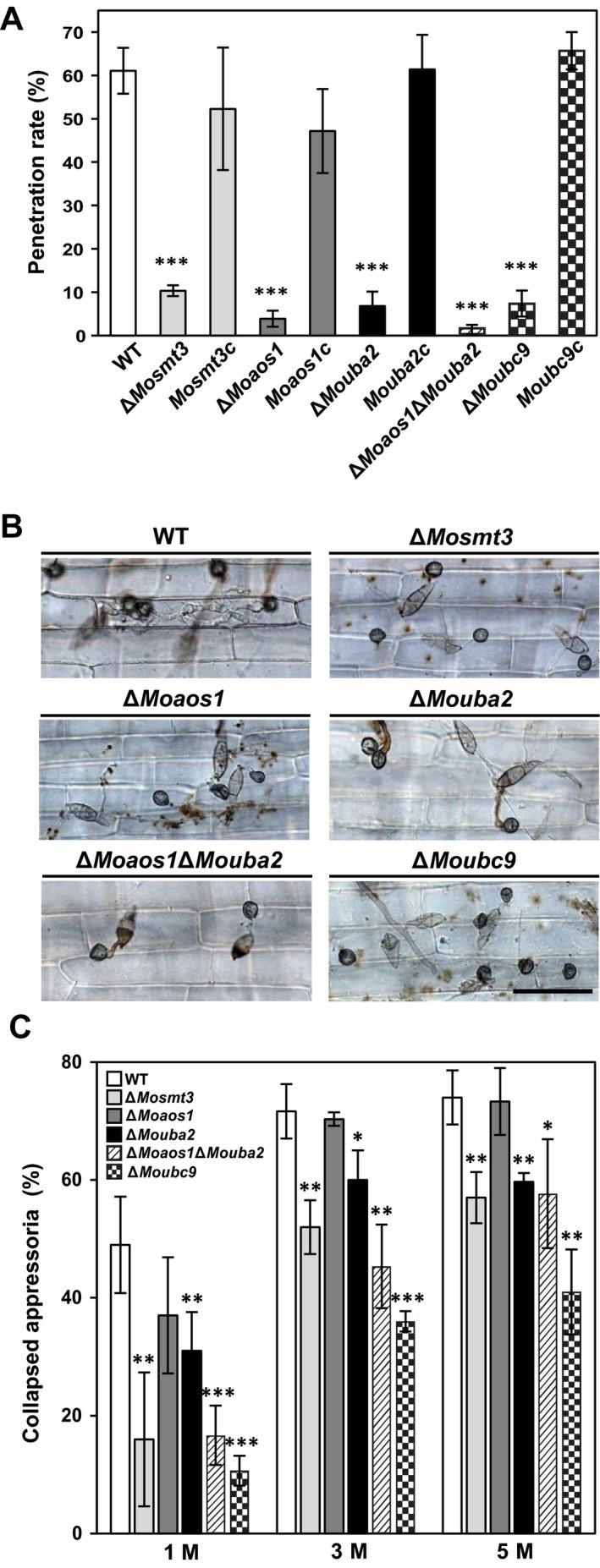
Penetration function of appressoria. (A, B) Rate of penetration of rice sheath cells by appressoria at 24 h post‐inoculation (hpi). Scale bar, 50 μm. (C) The number of collapsed appressoria after treatment with 1, 3 and 5 m glycerol. Significance was determined by *t*‐test (*P < 0.01, **P < 0.05 and ***P < 0.001). WT, wild‐type.

SUMOylation is necessary for virulence in *Candida glabrata*, an opportunistic pathogen of bloodstream infections (BSIs) (Gujjula *et al*., 2016). To investigate whether SUMOylation is required for the pathogenicity of *M. oryzae*, we performed spray and sheath inoculation assays. Conidial suspensions (5 × 10^4^/mL) of the wild‐type and deletion mutants were sprayed onto 4‐week‐old rice plants of cultivar Nakdongbyeo. The wild‐type induced typical lesions, but the deletion mutants showed smaller and fewer lesions (Fig. 7A). The disease lesion type was evaluated on the basis of lesion size (Valent *et al*., 1991). In the wild‐type and complemented strains, the disease lesion type was 4, but the disease lesion type of deletion mutants was 1.5–2. The reduced pathogenicity of the deletion mutants was recovered in the complemented strains (Fig. 7A). Penetration sites of appressoria were observed in rice sheath cells at 24 h post‐inoculation (hpi) under a microscope. In comparison with the wild‐type, collapsed appressoria were frequently observed in the rice sheath cells inoculated by the deletion mutants. In the wild‐type, 62% of appressoria penetrated host cells, compared with 1%–10% in the deletion mutants. In particular, the penetration function of Δ*Moaos1*Δ*Mouba2* was reduced to the greatest extent (Fig. 8A,B). These results suggest that the reduced pathogenicity was a result of defective penetration of appressoria. Therefore, we performed a cytorrhysis test to estimate the turgor pressure in appressoria. Matured appressoria of the wild‐type and deletion mutants were treated with glycerol solution (1, 3 and 5 m). In the wild‐type, the number of collapsed appressoria increased from 49% to 74% with increasing glycerol concentration. However, the Δ*Mosmt3*, Δ*Mouba2*, Δ*Moaos1*Δ*Mouba2* and Δ*Moubc9* mutants showed fewer collapsed appressoria than the wild‐type. (Fig. 8C). These data suggest that the turgor pressure in the appressoria of the deletion mutants was not less than that in the wild‐type. Therefore, to determine the cell wall integrity of appressoria, we performed plasmolysis assay using polyethylene glycol (PEG) of different molecular sizes. This showed that the plasmolysis to cytorrhysis ratios of the deletion mutants did not differ significantly from that of the wild‐type (data not shown).

Next, we performed a sheath inoculation assay to evaluate the cause of restricted lesion development on rice cells. Growth of invasive hyphae (IH) at 48 hpi was classified into three types: Type I for IH restricted to the primary infection cell, Type II for IH growth to adjacent cells and Type III for extensive growth of IH over adjacent cells. Although 82% of IH from the appressoria of the wild‐type were Type II and III, the corresponding values in the deletion mutants were only 31%–43% (Fig. S8B, see Supporting Information). Remarkably, IH growth was significantly reduced in Δ*Moaos1*Δ*Mouba2* compared with the single‐gene deletion mutants (Figs 7B and S8B). The reduced penetration rate and invasive growth were recovered in the complemented strains (Figs S8A and S9, see Supporting Information). These results indicate that SUMOylation is important for functional appressoria and the pathogenicity of *M. oryzae*.

### SUMOylation‐associated proteins are localized to the nucleus and cytoplasm

**Figure 9:**
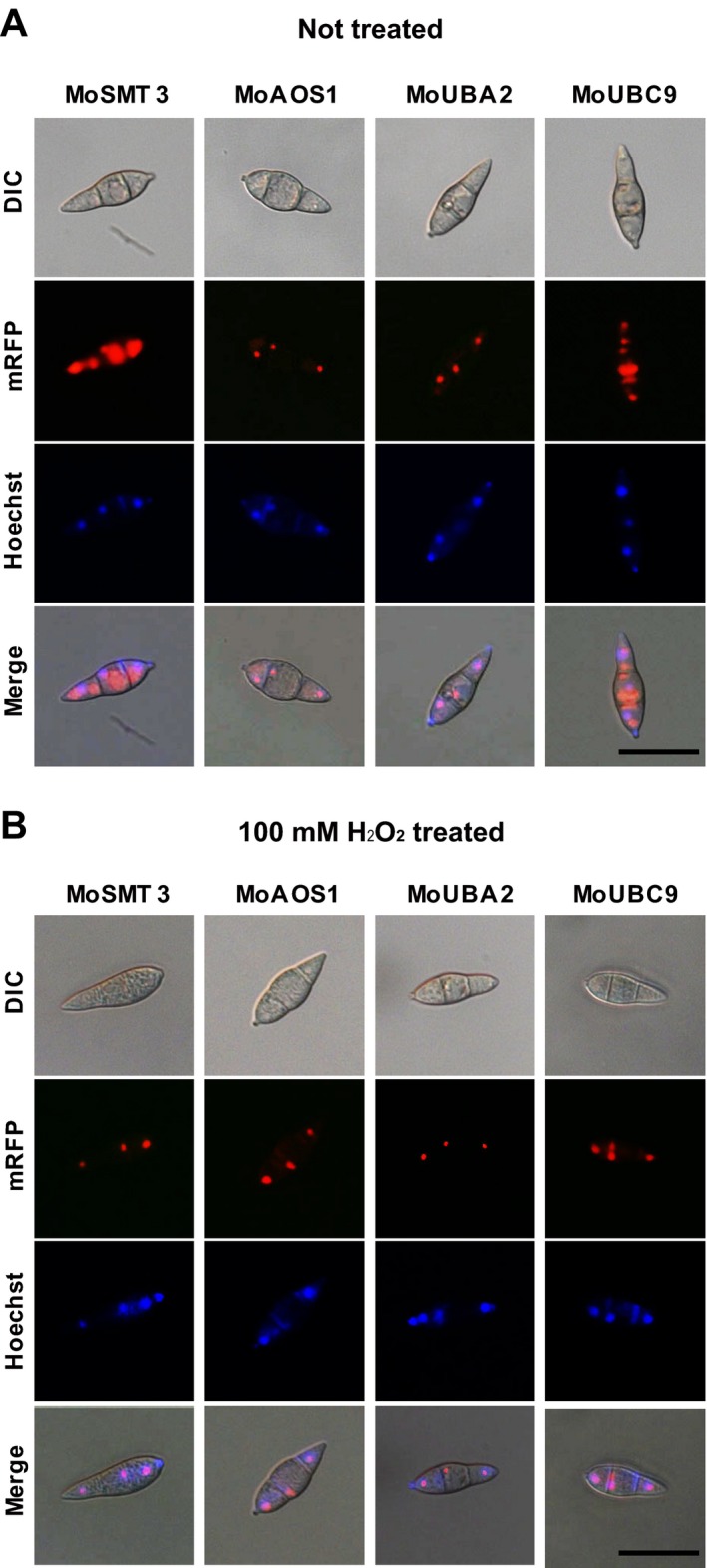
Intracellular localization of SUMOylation components in *Magnaporthe oryzae*. (A) MoAOS1 and MoUBA2 were localized predominantly in the nucleus, but MoSMT3 and MoUBC9 were localized to both the nucleus and the cytoplasm. Nuclei were stained with Hoechst 33342. Scale bar, 25 μm. (B) The four SUMOylation components were predominantly localized to the nucleus under oxidative stress conditions (100 mm H_2_O_2_). Scale bar, 25 μm. Differential interference contrast (DIC), mRFP, monomeric red fluorescent protein.

Most SUMOylation components are localized to the nucleus and, to a lesser extent, to the cytoplasm (Gill, 2004; Gillies *et al*., 2016; Truong *et al*., 2012). To observe the localization of SUMOylation components, we transformed the deletion mutants with plasmids containing monomeric red fluorescent protein (mRFP) fused with MoSMT3, MoAOS1, MoUBA2 or MoUBC9. Intracellular localization of MoSMT3, MoAOS1, MoUBA2 and MoUBC9 was observed under a fluorescence microscope. MoAOS1 and MoUBA2 were largely localized to the nucleus, but MoSMT3 and MoUBC9 were present in the nucleus and cytoplasm (Fig. S10, see Supporting Information). However, all four SUMOylation components were predominantly localized in the nucleus under oxidative stress conditions (100 mm H_2_O_2_) (Fig. 9B).

## Discussion

Since the discovery of SUMO in *S*. *cerevisiae* in 1995, SUMOylation has been investigated for over 20 years in other model organisms (Broday *et al*., 2004; Ihara *et al*., 2008; Meluh and Koshland, 1995; Nacerddine *et al*., 2005; Nie *et al*., 2009; Nowak and Hammerschmidt, 2006; Park *et al*., 2011). However, the machinery and functional roles of SUMOylation in plant‐pathogenic fungi are unclear. Recent advances in genome sequencing and molecular tools have enabled the identification and functional characterization of SUMOylation components. We comprehensively analysed SUMOylation in the model fungal plant pathogen *M. oryzae*.

### The SUMOylation machinery in fungi differs from other UBLs

An understanding of the function of the SUMOylation machinery is limited to model species. Unlike ubiquitination, SUMOylation does not involve protein degradation, but instead enhances protein stability (Müller *et al*., 2001). A previous study on ubiquitination in *M. oryzae* identified SUMO, E1 and E2 enzymes as ubiquitination components using a domain search method (Oh *et al*., 2012). However, SUMOylation and other PTMs in yeast utilize different machineries (Johnson *et al*., 1997; Müller *et al*., 2001). Our phylogenetic analysis showed that MoAOS1 and MoUBA2 are clearly separated from the other E1 UBLs. The ubiquitin E2 enzyme (MGG_01756) in *M. oryzae* (Shi *et al*., 2016) was not grouped with either MoUBC9 or ScUBC9, therefore, ubiquitination‐related proteins formed their own clade. In addition, the *MoSTM3*, *MoAOS1*, *MoUBA2* and *MoUBC9* deletion mutants exhibited normal ubiquitination. Although all UBLs have the same ubiquitin‐like domains, their diverse functions may be a result of differences in their sequences. For example, the three‐dimensional structure of human SUMO protein (12 kDa) is similar to that of ubiquitin (8.5 kDa) despite their 18% sequence similarity, and SUMO has an additional 15 amino acids at its N‐terminal end (Melchior, 2000; Müller *et al*., 2001). Overall, our data indicated that SUMOylation components are not involved in ubiquitination, instead being involved in a unique PTM.

### SUMOylation does not affect viability, but is essential for the development of *M. oryzae*

*Saccharomyces cerevisiae* deletion mutants Δ*smt3*, Δ*aos1*, Δ*uba2* and Δ*ubc9* were non‐viable (Schwarz *et al*., 1998). However, deletion of *MoSmt3*, *MoAos1*, *MoUba2* and *MoUbc9* did not affect the viability of *M. oryzae*. SUMO deletion mutants of *A. nidulans*, *A. flavus*, *C. albicans* and *S. pombe* were also viable (Leach *et al*., 2011; Nie *et al*., 2016; Shayeghi *et al*., 1997; Tanaka *et al*., 1999; Wong *et al*., 2008). Although fungi with deletions of SUMOylation‐associated genes are viable, they are defective in development. This phenomenon may be a result in part of the presence of structural paralogues of the SUMOylation‐associated proteins. Our phylogenetic analysis showed that the AOS1 clade contains only one protein in *S. cerevisiae*, but two proteins in other fungal species, such as *M. oryzae*, *A. nidulans* and *S. pombe*. This additional gene may have the same function as AOS1 for SUMOylation. However, this speculation does not fully support the viability of other SUMOylation‐defective mutants of *M. oryzae*, such as Δ*Mosmt3*. Alternatively, this may be explained by the ‘centrality–lethality’ rule (Jeong *et al*., 2001). Further study is required to understand the non‐lethality of SUMOylation‐defective mutants, including in fungi such as *M. oryzae*.

Like the other mutants of SUMOylation components in other organisms (Gujjula *et al*., 2016; Leach *et al*., 2011; Nie *et al*., 2016; Wong *et al*., 2008), Δ*Mosmt3*, Δ*Moaos1*, Δ*Mouba2*, Δ*Moaos1*Δ*Mouba2* and Δ*Moubc9* showed significantly reduced mycelial growth, conidiation, conidial germination, appressorium formation, stress resistance and pathogenicity. Deletion of the SUMOylation‐associated genes was not lethal, unlike in *S. cerevisiae* and *C. glabrata* (Gujjula *et al*., 2016; Schwarz *et al*., 1998), but deletion of even one of these genes almost abolished SUMOylation in *M. oryzae*. These data indicate that all SUMOylation components are indispensable for SUMOylation. The pleiotrophic phenotypes of the deletion mutants may be a result of the dysfunction of SUMOylation, because SUMOylated proteins play roles in chromatin organization, transcriptional regulation, RNA biosynthesis and ribosome biogenesis (Nie *et al*., 2015).

### SUMOylation is involved in conidiation

Conidiation is a key factor in the outbreak of rice blast epidemics (Kim *et al*., 2009). In this study, conidiation was defective in the SUMOylation‐associated gene deletion mutants. However, the expression of conidiation‐related genes (*Cos1*, *Com1*, *Con7* and *Hox2*), reported by others (Kim *et al*., 2009; Odenbach *et al*., 2007; Yang *et al*., 2010; Zhou *et al*., 2009), was not significantly increased or decreased in the deletion mutants. These data suggest that conidiation is regulated not only at the transcriptional level, but also at the post‐translational level. This is supported by the fact that SUMO acts as a stabilizer of transcription factors (Rosonina *et al*., 2017). Subsequent identification of SUMOylated motifs in conidiation‐related genes further supports the hypothesis that the modification of conidiation‐related genes by SUMO controls conidiation in *M. oryzae*. Similar conidiation phenotypes were reported in SUMOylation mutants of *A. nidulans* and *A. flavus* (Nie *et al*., 2016; Wong *et al*., 2008). These results suggest that SUMOylation is crucial for conidiation in filamentous fungi.

### SUMOylation provides new insights into the function of appressoria and pathogenicity

In this study, spray inoculation of the deletion mutants resulted in the generation of fewer and smaller lesions. The rates of penetration by appressoria of the deletion mutants were significantly reduced, suggesting the dysfunctionality of appressoria. A cytorrhysis assay indicated that the turgor pressure generation in the appressoria of deletion mutants was abnormal, but not less than that of the wild‐type. Furthermore, ratios of plasmolysis to cytorrhysis, indicating the cell wall integrity, were not significantly different between the deletion mutants and the wild‐type. Remodelling of the actin cytoskeleton, together with turgor pressure and cell wall integrity, has been reported as another important factor for appressoria function (Dagdas *et al*., 2012). Deletion mutants of septin GTPase showed defects in penetration and pathogenicity, because of the abnormal assembly of the actin cytoskeleton, in *M. oryzae* (Gupta *et al*., 2015). These septin proteins, MoSEP3, MoSEP4, MoSEP5 and MoSEP6, are predicted to be SUMOylated according to GPS‐SUMO 2.0 (Zhao *et al*., 2014). This suggests that SUMOylation may affect other factors, such as remodelling of the actin cytoskeleton, rather than turgor generation and the cell wall integrity of appressoria.

IH growth of the deletion mutants was significantly lower than that of the wild‐type. This may be a result of the role of SUMOylation conferring tolerance to oxidative stress induced by the plant (Feligioni and Nisticò, 2013). The degree of inhibition of mycelial growth of the deletion mutants by oxidative stress was greater than that of the wild‐type. Further study should evaluate the mechanism by which SUMOylation affects oxidative stress resistance and IH growth.

In this study, we found that the SUMOylation machinery differs from that involved in ubiquitination, and evaluated the roles of SUMOylation‐associated genes in infection‐related development, the stress response and the pathogenicity of *M. oryzae*. These findings provide not only new insights into SUMOylation as a unique PTM in fungi, but also facilitate the identification of the mechanisms of pathogenesis of *M. oryzae* and other fungal plant pathogens.

## Experimental Procedures

### Identification of SUMOylation machinery in fungi

SUMOylation components in fungi and model organisms were identified by whole‐protein blast search of the *S. cerevisiae* SUMO (SMT3), E1 (AOS1 and UBA2), E2 (UBC9), E3 (SIZ1, SIZ2, MMS21 and CST9) and SUMO protease (ULP1, ULP2 and WSS1) sequences using an e‐value of 1e^−5^. The fungal proteome datasets used were the Fungal Genome Gold Standard from CFGP 2.0 (Choi *et al*., 2013), the model organisms were from Ensembl (Aken *et al*., 2016) and the *S. cerevisiae* query proteins were from Saccharomyces Genome Database (SGD, https://www.yeastgenome.org/) (Cherry, 2015). A domain analysis was performed using InterProScan‐5.25–64.0 software to eliminate sequences without characterized domains. The domain sequences were retrieved and aligned by MAFFT software (Katoh and Standley, 2013) and sparsely aligned regions were trimmed using trimAl software (Capella‐Gutierrez *et al*., 2009). For phylogenetic analysis, the best protein evolution model for each component alignment was determined, and phylogenetic trees were constructed using RAxML v8.2 software with 1000 bootstraps (Stamatakis, 2014).

### Targeted gene deletion and complementation

*Magnaporthe oryzae* KJ201 (wild‐type) was obtained from the Center for Fungal Genetic Resources (CFGR) at Seoul National University, Seoul, South Korea (Table S6, see Supporting Information). Protoplasts were generated from mycelia grown in liquid complete medium (CM) at 25 °C for 3 days. The upstream (1.0–1.5 kb) and downstream (1.0–1.5 kb) flanking regions of each SUMOylation‐associated gene (*MoSMT3*, *MoAos1*, *MoUba2* and *MoUbc9*) were amplified from the genomic DNA (gDNA) of the wild‐type (Table S7, see Supporting Information). The 1.4‐kb hygromycin B phosphotransferase gene (HPH) cassette was amplified from pBCATPH (Yun, 1998). Constructs were produced by double‐joint PCR using three amplicons (upstream flanking, downstream flanking and HPH cassette) for targeted gene deletion. Targeted gene deletion mutants were generated by transforming the constructs into protoplasts using PEG. The targeted gene deletion mutants were selected on TB3 agar supplemented with 200 ppm hygromycin B, and screened by PCR analysis of mycelia using the open reading frame (ORF) primers. To generate the double‐gene deletion mutant, a 1.2‐kb geneticin resistance cassette was amplified from pII99 (Lee *et al*., 2003), and transformed into Δ*Moaos1* protoplasts. To produce complementation strains, constructs containing each ORF and promoter were amplified using upstream forward and downstream reverse primers for the gDNA of the wild‐type. The complemented strains were generated by co‐transformation of these constructs and a geneticin cassette into protoplasts of the single‐gene deletion mutants. The complemented strains were selected on TB3 agar supplemented with 800 ppm geneticin, and screened by PCR of mycelia using the ORF primers. All strains used in this study have been deposited in the Center for Fungal Genetic Resources (http://genebank.snu.ac.kr) at Seoul National University, Seoul, South Korea.

### Southern blotting and RT‐PCR

The wild‐type and deletion mutants were cultured in liquid CM at 25 °C for 3 days to extract gDNA and total RNA, as described previously (Kong *et al*., 2015). The gDNA was digested at 37 °C with restriction enzymes, separated on a 1% agarose gel and transferred to a nitrocellulose membrane. The membrane was hybridized with p32‐labelled probe generated using a Random Primers DNA Labeling System kit (Invitrogen, California, USA), and exposed to X‐ray film. Total RNA (5 μg) was reverse transcribed to complementary DNA (cDNA) using an ImProm‐II Reverse Transcription System kit (Promega, Wisconsin, USA), and subjected to qRT‐PCR using 50 ng of cDNA, 3 μL of primers and 5 μL of SYBR Green PCR Master Mix in a Rotor‐Gene Q 2plex (Qiagen, Hilden, Germany). Transcript levels were quantified by RT‐PCR using 100 ng of cDNA, 3 μL of primers and 10 μL of 2X master mix in a C1000 thermal cycler (Bio‐Rad, California, USA).

### Mycelial growth, conidiation and appressorium formation

All strains were cultured in modified complete agar medium (CMA) at 25 °C for 9 days for assessment of mycelial growth and colony morphology (Iyer and Chattoo, 2003). Conidia were collected from cultures on V8 agar after incubation for 7 days, and conidiation was measured under a microscope using a haemacytometer. To assess conidial germination and appressorium formation, 70 μL of conidial suspension (3 × 10^4^/mL) was dropped onto a hydrophobic cover glass at 25 °C. Conidial germination and appressorium formation were evaluated under a microscope after incubation for 2, 8 and 24 h. Conidiogenesis and conidial adhesion assays were performed as described previously (Goh *et al*., 2011a; Lau and Hamer, 1998). These experiments were performed in triplicate three times.

The morphology of conidia was observed using at least 100 conidia. Hyphae from conidia were incubated with CFW (10 μg/mL; Sigma Aldrich, Missouri, USA) at 25 °C for 5 min to stain the septa, and visualized using a fluorescence microscope (Carl Zeiss, Oberkochen, Germany). Cell length was measured using ImageJ software. These experiments were performed in triplicate three times.

### Cytorrhysis and plasmolysis assays

Conidial suspensions (3 × 10^4^/mL) was incubated on a hydrophobic cover glass at 25 °C for 48 h. The sterilized distilled water (SDW) was removed, and the appressoria were treated with glycerol (1, 3 and 5 m) or PEG (400, 1000, 3350 and 8000 Da). At least 100 collapsed and plasmolysed appressoria were counted under a microscope. These experiments were performed in triplicate three times.

### Sensitivity assessment to stress conditions

MMA was used to test sensitivity to nutrient starvation stress (Choi *et al*., 2015). To assess sensitivity to DNA damage stress, CMA was supplemented with 10 mm HU (DNA synthesis inhibitor) and 0.05% MMS (DNA alkylating agent). For oxidative stress, CMA was supplemented with 5 mm H_2_O_2_ and 3 mm MV. The wild‐type, deletion mutants and complemented strains were cultured in triplicate under the above five stress conditions and CMA (control) at 25 °C for 9 days.

### Pathogenicity tests

A pathogenicity test was performed using the susceptible rice cultivar Nakdongbyeo. For spray inoculation assays, 10 mL of conidial suspension (5 × 10^4^/mL, containing 250 ppm Tween‐20) was inoculated onto 4‐week‐old rice seedlings. Inoculated rice plants were incubated at 25 °C for 1 day at a relative humidity (RH) of 100% in the dark, and then at 28 °C for 5–6 days in a growth chamber. For sheath inoculation assays, conidial suspension (2 × 10^4^/mL) was inoculated onto sheaths of 6‐week‐old rice seedlings, which were then incubated at 25 °C for 24 or 48 h. At least 50 infection sites were observed under a microscope. These experiments were performed in triplicate three times.

### Western blot

The promoters of *MoSmt3* (*Eco*RI‐*Sal*I) and HA‐fused *MoSmt3* (*Sal*I‐*Apa*I) were thymine and adenine (TA) cloned into pGEMT‐easy (Promega) (Table S7). The cloned vectors were digested with the corresponding restriction enzymes and ligated into pCB1004 (Wang *et al*., 1999). The pCB1004:native promoter:HA:SMT3 (pHA‐SMT3) and geneticin resistance cassette were co‐transformed into the wild‐type and single‐gene deletion mutants. Transformants were cultured in liquid CM at 25 °C for 3 days to harvest the mycelia. Proteins were extracted using 600 μL of PRO‐PREP protein extraction solution (Intron Biotechnology, Seongnam, South Korea). Proteins (75 μg) were separated on a 4%–20% gradient SDS‐PAGE gel (Bio‐Rad) and transferred to an Immun‐Blot LF Polyvinylidene difluoride (PVDF) membrane using transfer buffer [20% methanol (v/v), 0.25 m Tris and 2 m glycine). The membrane was probed with an anti‐HA antibody (1 : 1000, Bethyl, Alabama, USA) and anti‐ubiquitin antibody (P4D1) (1 : 1000, Cell Signaling Technology, Massuchusetts, USA) using a Pierce Fast Western Blot Kit and ECL Substrate (Pierce Biotechnology, Rockford, IL, USA). An anti‐actin antibody (1 : 1000, Cell Signaling Technology) was used as the loading control. The probed membrane was exposed to X‐ray film.

### Y2H assay

The coding sequences of MoAOS1 and MoUBA2 were amplified from cDNA of the wild‐type (Table S7). A Y2H assay was performed using the ProQuest Two‐Hybrid System kit (Invitrogen). The coding sequences of MoAOS1 and MoUBA2 were inserted into both pDEST22 and pDEST32 using a Gateway system. The MaV203 yeast strain was cultured in Yeast extract‐Peptone‐Adenine‐Dextrose (YPAD) liquid medium at 30 °C until an optical density at 600 nm of 0.4 was reached (Table S6). Yeast competent cells were generated using lithium acetate (LiAc). Next, 1 μg of *MoAOS1* and *MoUBA2* inserted vectors, 100 μg of denatured salmon sperm DNA and 100 μL of competent cells were co‐transformed using LiAc and PEG. The control plasmids (pEXP22RalGDS‐wt, pEXP22RalGDS‐m1 and pEXP22RalGDS‐m2) were co‐transformed with pEXP32Krev1 using the same method and selected on SC‐Leu/Trp (SC‐LT) agar. Selected co‐transformants were confirmed by amplifying the inserted coding sequence. Interaction of MoAOS1 and MoUBA2 was confirmed on SC‐Leu/Trp/His + 25 mm 3AT (SC‐LTH + 3AT) agar and validated by X‐gal assay.

### Intracellular localization

The EF1α promoter (originating from *Fusarium verticillioides*) was amplified from YL1320. The coding sequences of MoSMT3, MoAOS1, MoUBA2 and MoUBC9 were amplified from the cDNA of the wild‐type. Fusion constructs of the EF1α promoter and each coding sequence were generated using double‐joint PCR and fused in pFPL‐rh containing the coding sequence of mRFP. Next, the coding sequence of mRFP was conjugated to the C‐terminal coding sequence. The C‐terminus of SMT3 is cleaved on reaching maturity. Therefore, another plasmid was generated to observe the localization of SMT3. mRFP and the TrpC terminator were amplified from pFPL‐rh and pBCATPH. The EF1α promoter:mRFP (*Xba*I‐*Hin*dIII) and SMT3:TrpC terminator (*Hin*dIII‐*Xho*I) fusion constructs were generated using double‐joint PCR and TA cloned into pGEMT‐easy. The cloned vectors were digested with the corresponding restriction enzymes and fused in pCB1004. The pFPL‐rh:EF1α promoter:AOS1 (pLOCAL01669), pFPL‐rh:EF1α promoter:UBA2 (pLOCAL06733), pFPL‐rh:EF1α promoter:UBC9 (pLOCAL00970) and pCB1004:EF1α promoter:mRFP:SMT3:TrpC terminator (pLOCAL05737) constructs were co‐transformed with a geneticin resistance cassette into all of the deletion mutants. The localization of MoSMT3, MoAOS1, MoUBA2 and MoUBC9 in conidia and developmental stages, including conidial germination and appressorium formation, was observed using a fluorescence microscope (Carl Zeiss). Conidia were incubated in Hoechst33342 solution (10 mg/mL, Invitrogen) at 25 °C for 30 min to stain the nuclei.

## Supporting information

Additional Supporting Information may be found in the online version of this article at the publisher's website:

**Fig. S1** Phylogenetic trees of SUMOylation components in fungi and model organisms. Phylogenetic trees of SUMOylation components in fungi and model organisms that were homologous to *Saccharomyces cerevisiae* SUMOylation components were constructed by the maximum‐likelihood method with 1000 bootstraps and the LG or BLOSUM62 protein substitution method. The sequences used for alignments were: (A) PF11976 for SUMO, SMT3; (B) PF00899 for E1, AOS1 and UBA2; (C) PF00179 for E2, UBC9; (D) PF02891 for E3, SIZ1 and SIZ2; (E) PF11789 for E3, MMS21; (F) PF02902 for protease, ULP1 and ULP2; (G) PF08325 for protease and WSS1. Clades that contained more than three species of the same fungal phylum, animal and plant are shown without genus/species names. The yeast components are marked with a star and *Magnaporthe oryzae* homologues are shown in bold. Only bootstrap values >50% are shown.

**Fig. S2** Southern blot analysis and reverse transcription‐polymerase chain reaction (RT‐PCR) of the deletion mutants. Genomic DNA of wild‐type (WT) and deletion mutants was extracted and digested with *Pst*I, *Sal*I, *Hin*dIII or *Xho*I. The upstream or downstream construct of each gene was used as a probe for Southern blot analysis. Complementary DNA was synthesized from total RNA. β‐tubulin was used for normalization.

**Fig. S3** SUMOylation in wild‐type (WT) and Δ*Moaos1* during conidiation. Protein extract from the WT during conidiation was separated by sodium dodecylsulfate‐polyacrylamide gel electrophoresis (SDS‐PAGE) and subjected to Western blot analysis using an anti‐haemagglutinin (HA) antibody.

**Fig. S4** SUMOylated proteins in wild‐type (WT) under oxidative stress. Protein extract from the WT treated with oxidative stress (100 mm H_2_O_2_) was separated by sodium dodecylsulfate‐polyacrylamide gel electrophoresis (SDS‐PAGE) and subjected to Western blot analysis using an anti‐haemagglutinin (HA) antibody.

**Fig. S5** Ubiquitination in the wild‐type (WT), Δ*Mosmt3*, Δ*Moaos1*, Δ*Mouba2* and Δ*Moubc9*. Protein extracts of WT, Δ*Mosmt3*, Δ*Moaos1*, Δ*Mouba2* and Δ*Moubc9* were separated by sodium dodecylsulfate‐polyacrylamide gel electrophoresis (SDS‐PAGE) and subjected to Western blot analysis using an anti‐ubiquitin (P4D1) antibody. Ubiquitination was not affected in all of the deletion mutants.

**Fig. S6** Defective conidiogenesis was recovered in complemented strains. Conidiogenesis on conidiophores was observed under a microscope. Scale bar, 100 µm.

**Fig. S7** Mycelial growth under stress conditions. Strains were inoculated on nutrient starvation stress [minimal agar medium (MMA)], DNA damage stress [10 mm hydroxyurea (HU) and 0.05% methyl methanesulfonate (MMS)] and oxidative stress [5 mm H_2_O_2_ and 3 mm methyl viologen (MV)]. Mycelial growth was measured 9 days after inoculation. CMA, complete agar medium.

**Fig. S8** Growth of invasive hyphae (IH) in the complemented strains. (A) Conidial suspensions (2 × 10^4^/mL) of the complemented strains were inoculated onto 6‐week‐old rice sheath cells. IH growth was observed under a microscope at 48 h post‐inoculation (hpi). Scale bar, 50 μm. (B) Growth of IH into neighbouring cells was classified into three types: Type I for IH restricted to the primary infection cell; Type II for IH growth to adjacent cells; Type III for extensive growth of IH over adjacent cells.

**Fig. S9** Penetration assay of the complemented strains. Conidial suspensions (2 × 10^4^/mL) of the complemented strains were inoculated onto 6‐week‐old rice sheath cells. Penetration by appressoria at 24 h post‐inoculation (hpi) was observed under a microscope. Scale bar, 50 μm.

**Fig. S10** Intracellular localization of SUMOylation components in *Magnaporthe oryzae*. MoAOS1, MoUBA2, MoSMT3 and MoUBC9 were predominantly localized in the nuclei during conidial germination and appressorium formation. Scale bar, 50 μm.

**Table S1** Domains of *Saccharomyces cerevisiae* SUMOylation components.

**Table S2** The number of *Saccharomyces cerevisiae* homologues of SUMOylation components in the selected species.

**Table S3** The closest *Saccharomyces cerevisiae* homologues of SUMOylation components in *Magnaporthe oryzae*.

**Table S4** Developmental phenotypes of the wild‐type (WT), deletion mutants and complemented strains.

**Table S5** Conidial germination and appressorium formation of the wild‐type (WT), deletion mutants and complemented strains after incubation for 2, 8 and 24 h.

**Table S6** List of the strains used in this study.

**Table S7** Primer sequences used in this study.
