# Supplementary material for: SUMOylation is required for fungal development and pathogenicity in the rice blast fungus Magnaporthe oryzae
Source: Mol Plant Pathol. 2018 Jul 17;19(9):2134–48. doi: 10.1111/mpp.12687 (PMC6638150; doi:10.1111/mpp.12687)

**Figure S1. Phylogenetic trees of SUMOylation components in fungi and model organisms.** Phylogenetic trees of SUMOylation components in fungi and model organisms that are homologous to *S. cerevisiae* SUMOylation components were constructed by the maximum-likelihood method with 1,000 bootstraps and the LG or BLOSUM62 protein substitution method. The sequences used for alignments were (A) PF11976 for SUMO, SMT3; (B) PF00899 for E1, AOS1, and UBA2; (C) PF00179 for E2, UBC9; (D) PF02891 for E3, SIZ1, and SIZ2; (E) PF11789 for E3, MMS21; (F) PF02902 for protease, ULP1, and ULP2; and (G) PF08325 for protease and WSS1. Clades that contained more than three species of the same fungal phylum, animal, and plant are shown without genus/species names. The yeast components are marked with star and *M. oryzae* homologs are shown in bold. Only bootstrap values > 50% are shown.


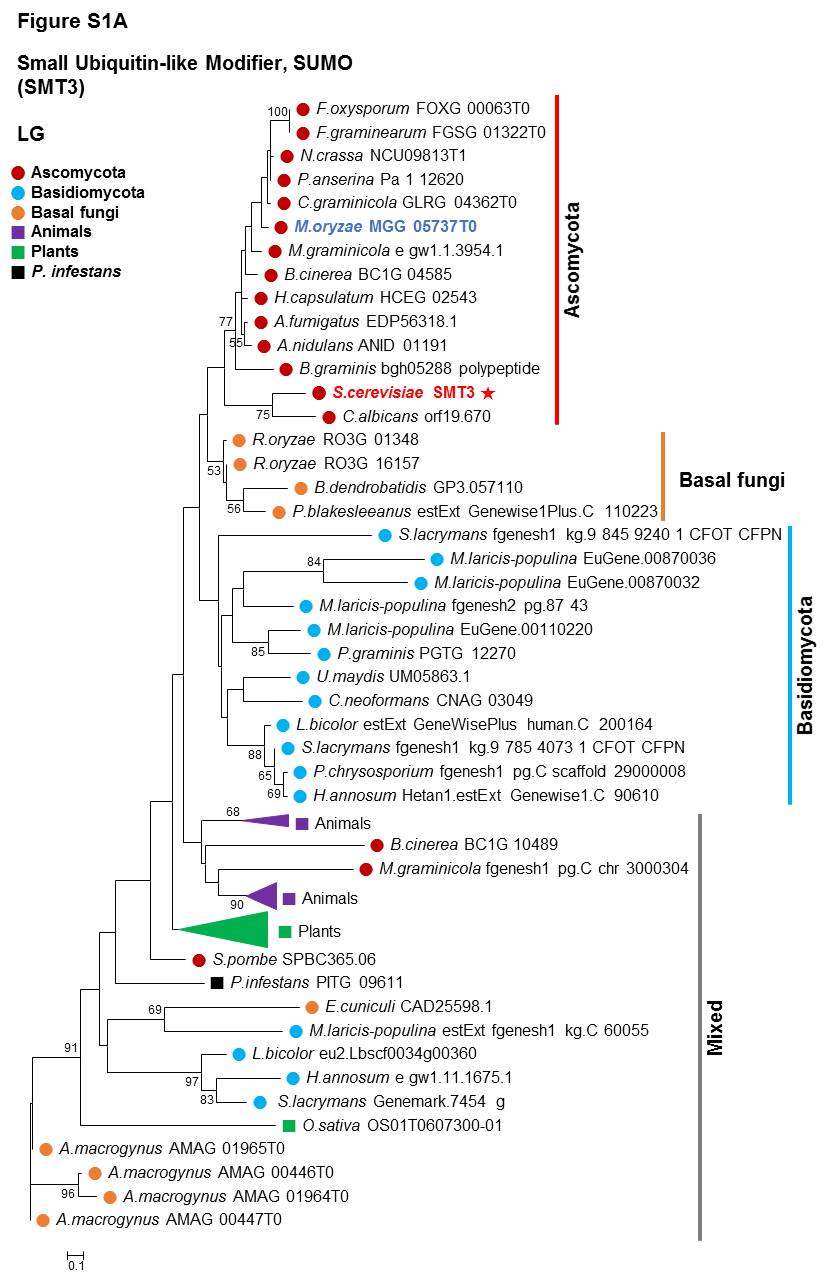


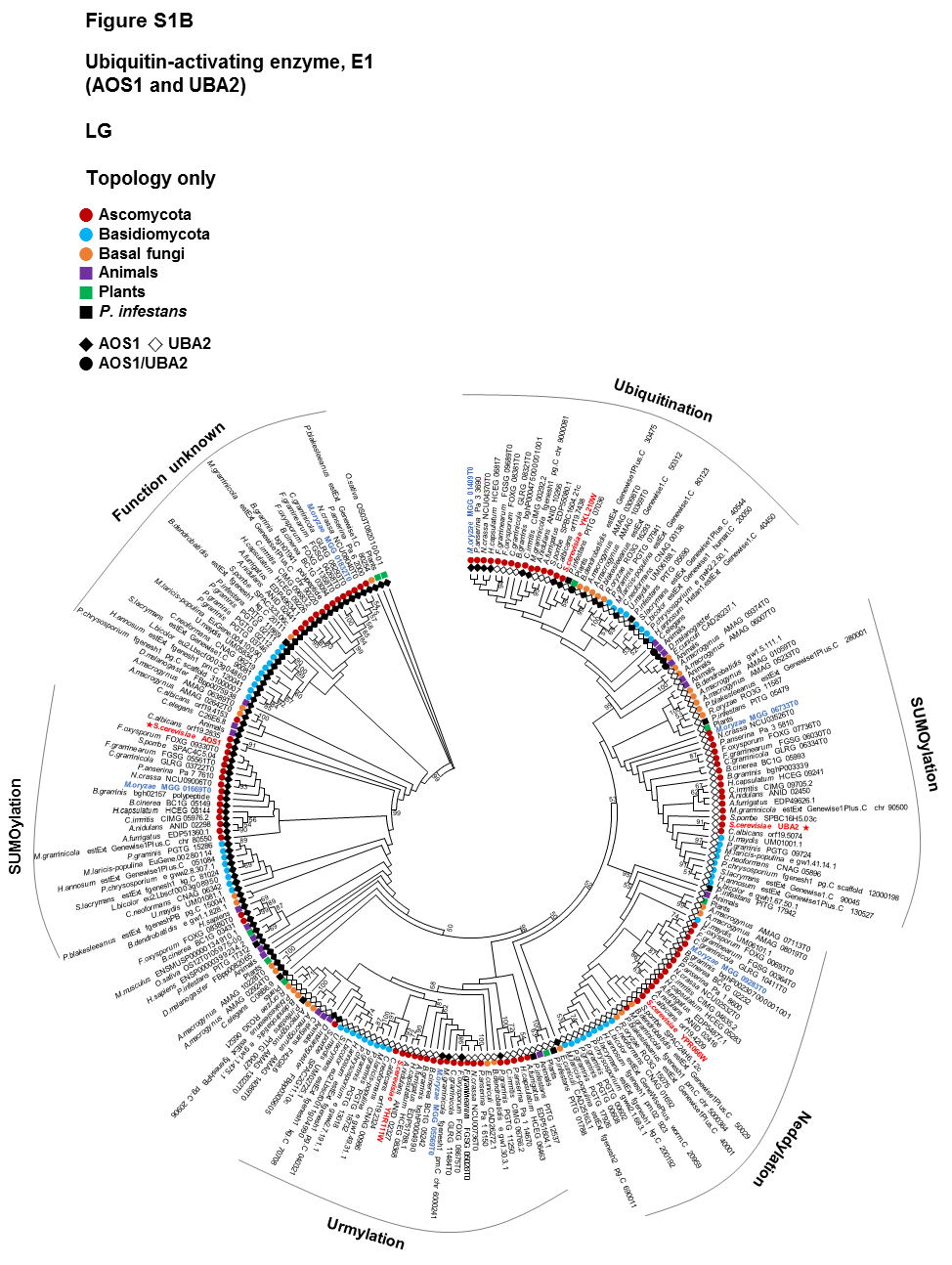


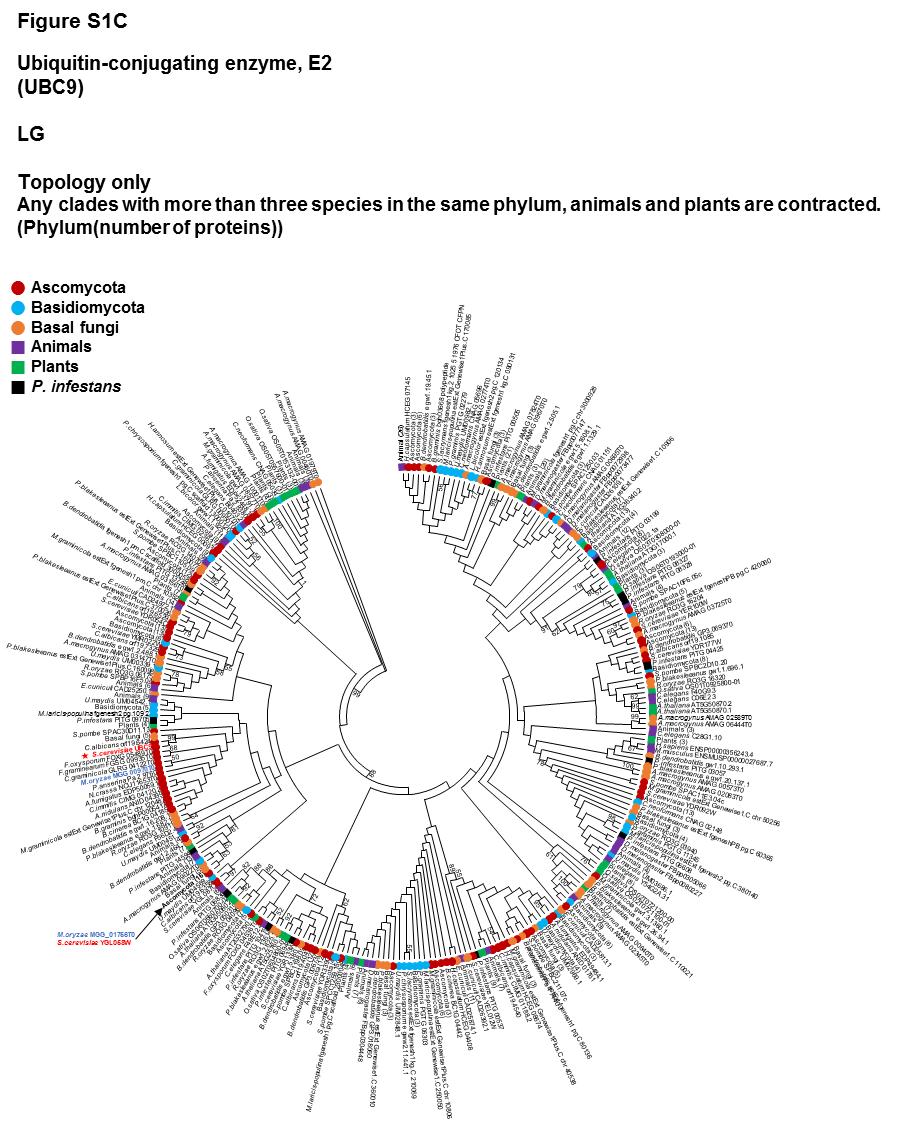


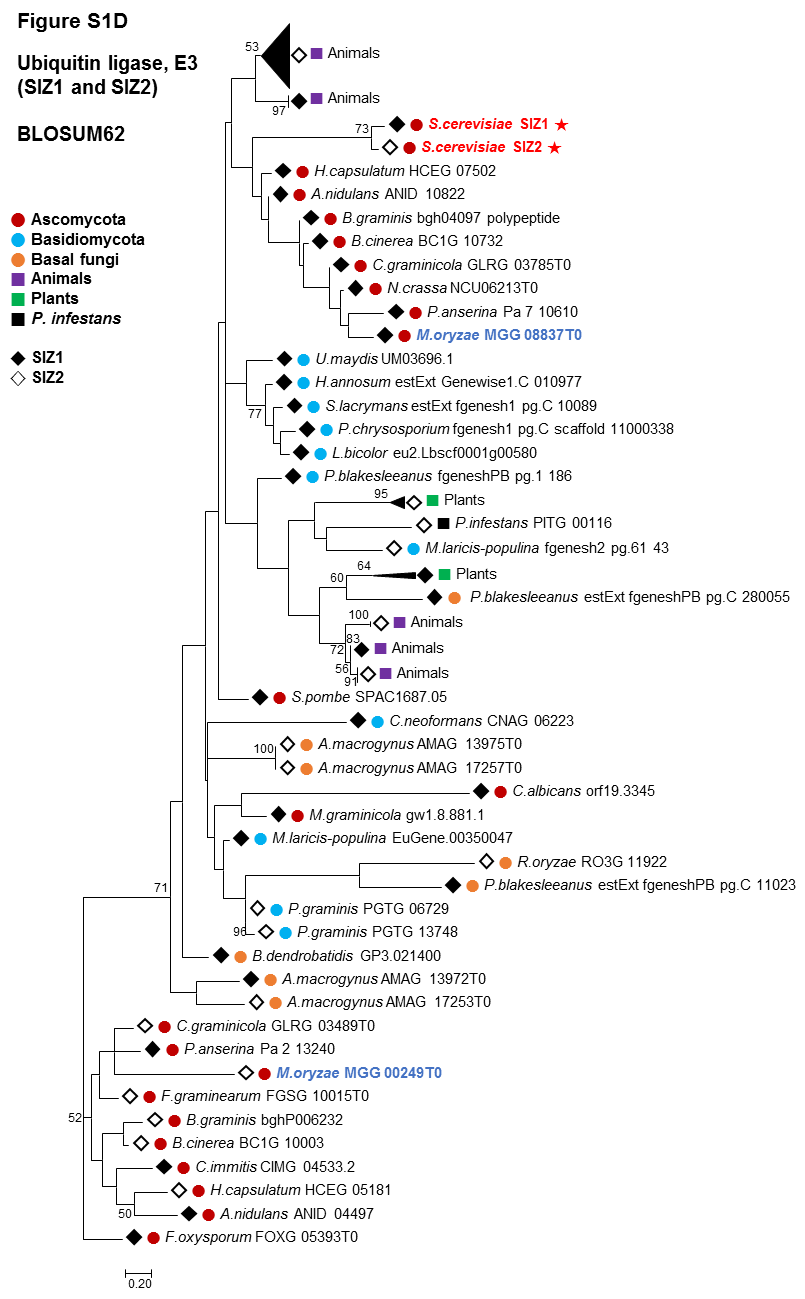


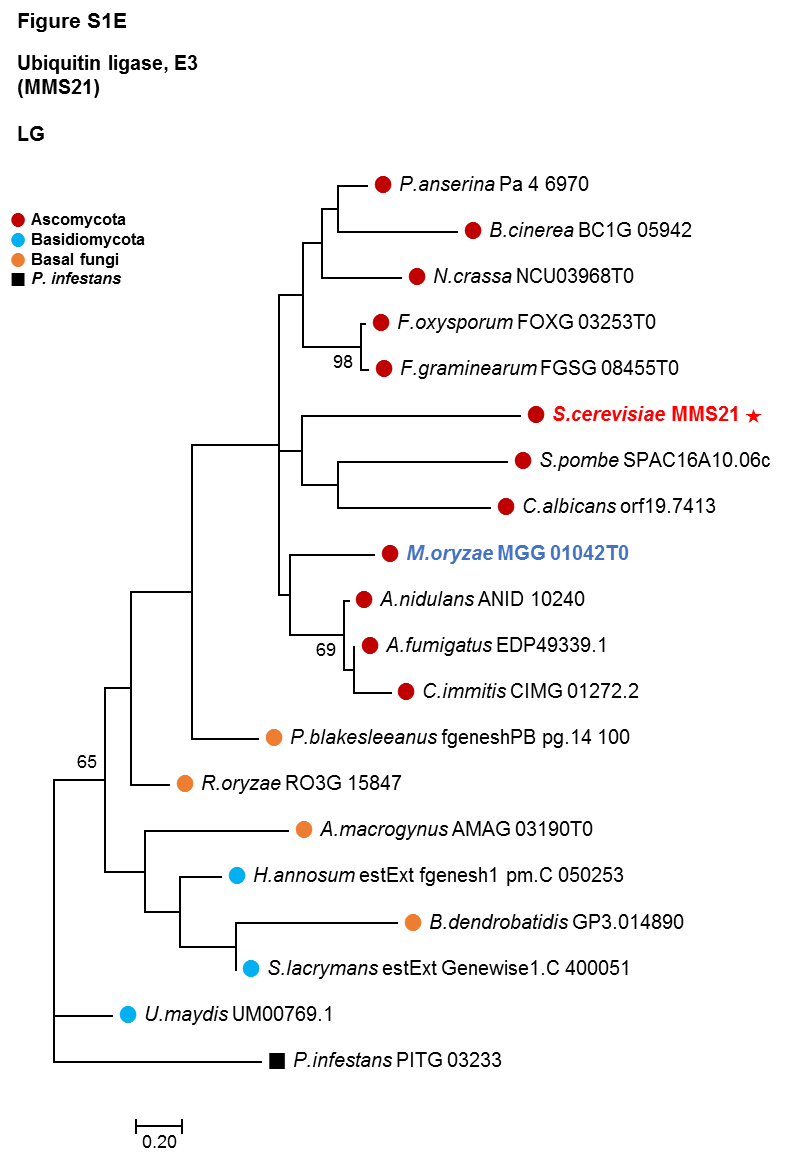


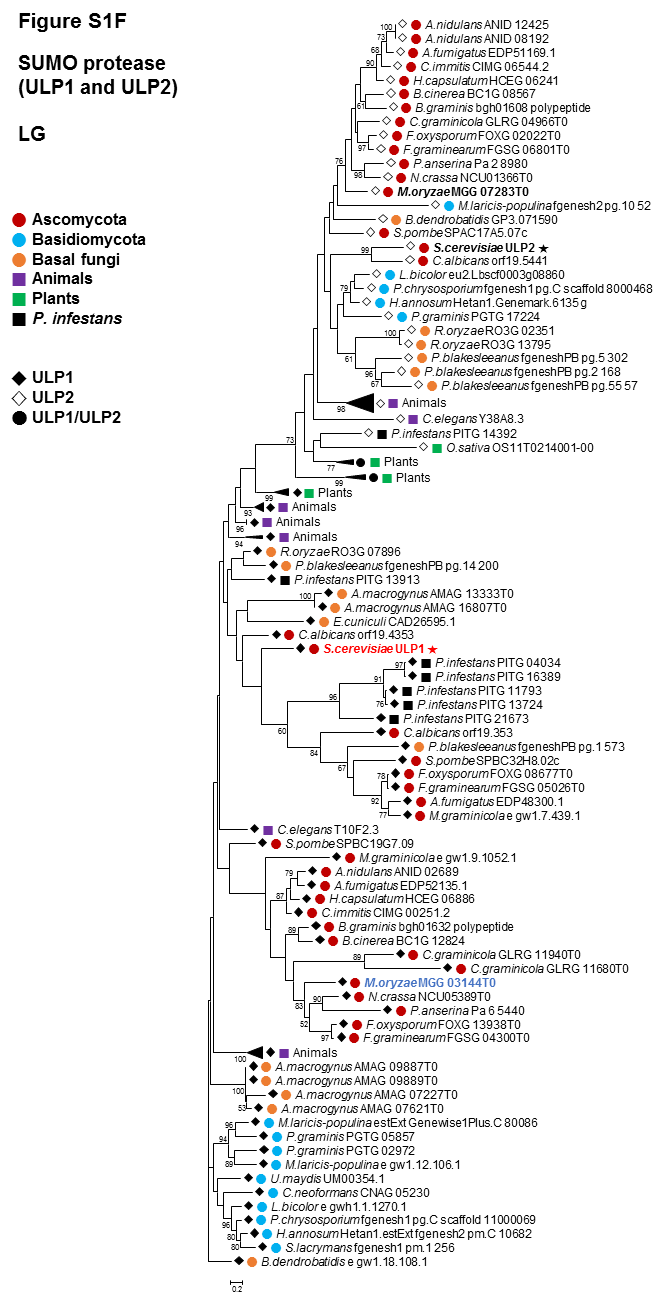


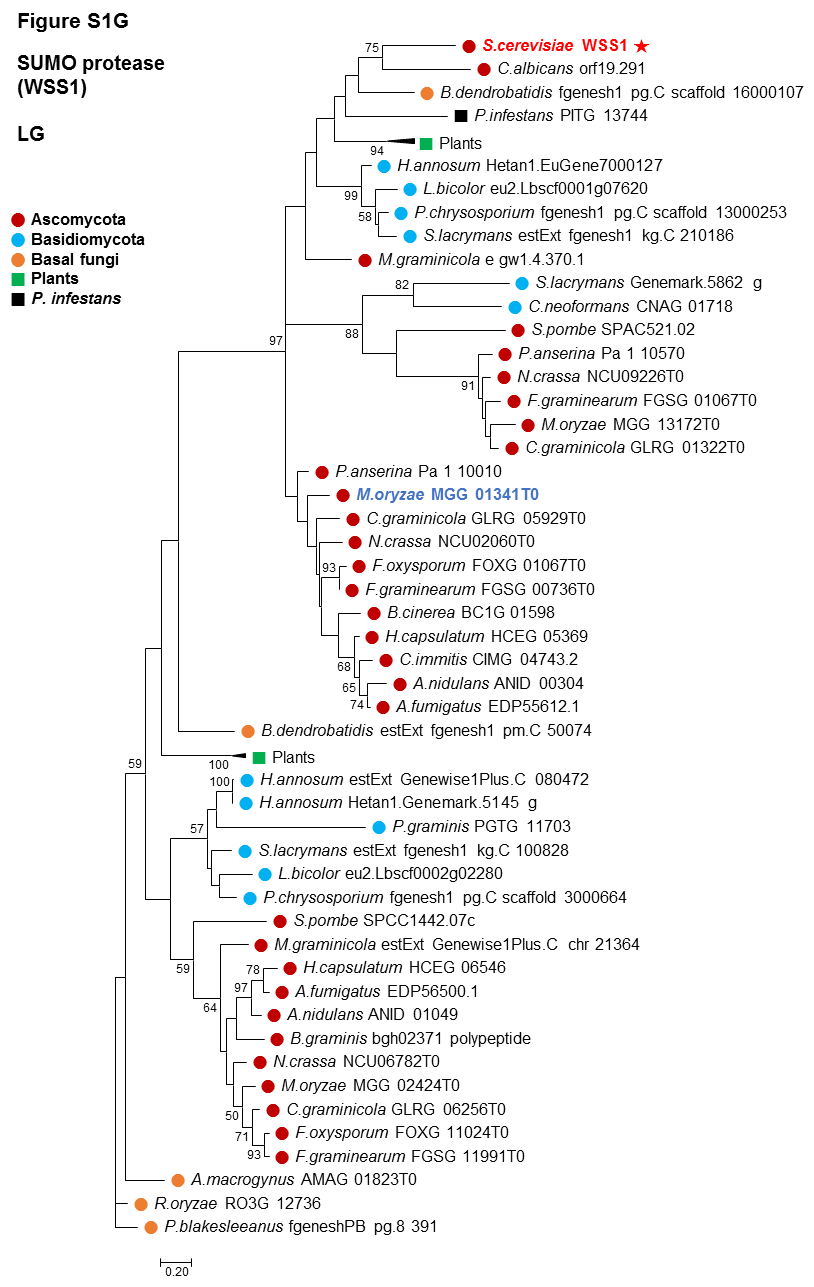

Supplement: Supplementary file 1 — Fig. S1 Phylogenetic trees of SUMOylation components in fungi and model organisms. Phylogenetic trees of SUMOylation components in fungi and model organisms that were homologous to Saccharomyces cerevisiae SUMOylation components were constructed by the maximum‐likelihood method with 1000 bootstraps and the LG or BLOSUM62 protein substitution method. The sequences used for alignments were: (A) PF11976 for SUMO, SMT3; (B) PF00899 for E1, AOS1 and UBA2; (C) PF00179 for E2, UBC9; (D) PF02891 for E3, SIZ1 and SIZ2; (E) PF11789 for E3, MMS21; (F) PF02902 for protease, ULP1 and ULP2; (G) PF08325 for protease and WSS1. Clades that contained more than three species of the same fungal phylum, animal and plant are shown without genus/species names. The yeast components are marked with a star and Magnaporthe oryzae homologues are shown in bold. Only bootstrap values >50% are shown. [file MPP-19-2134-s001.docx]
