# Supplementary material for: SUMOylation is required for fungal development and pathogenicity in the rice blast fungus Magnaporthe oryzae
Source: Mol Plant Pathol. 2018 Jul 17;19(9):2134–48. doi: 10.1111/mpp.12687 (PMC6638150; doi:10.1111/mpp.12687)

**Figure S2. Southern blot analysis and RT-PCR of the deletion mutants.** Genomic DNA of wild type and deletion mutants was extracted and digested with *Pst*I, *Sal*I, *Hin*dIII, or *Xho*I. The upstream or downstream construct of each gene was used as a probe for Southern blot analysis. Complementary DNA was synthesized from total RNA. β-tubulin was used for normalization


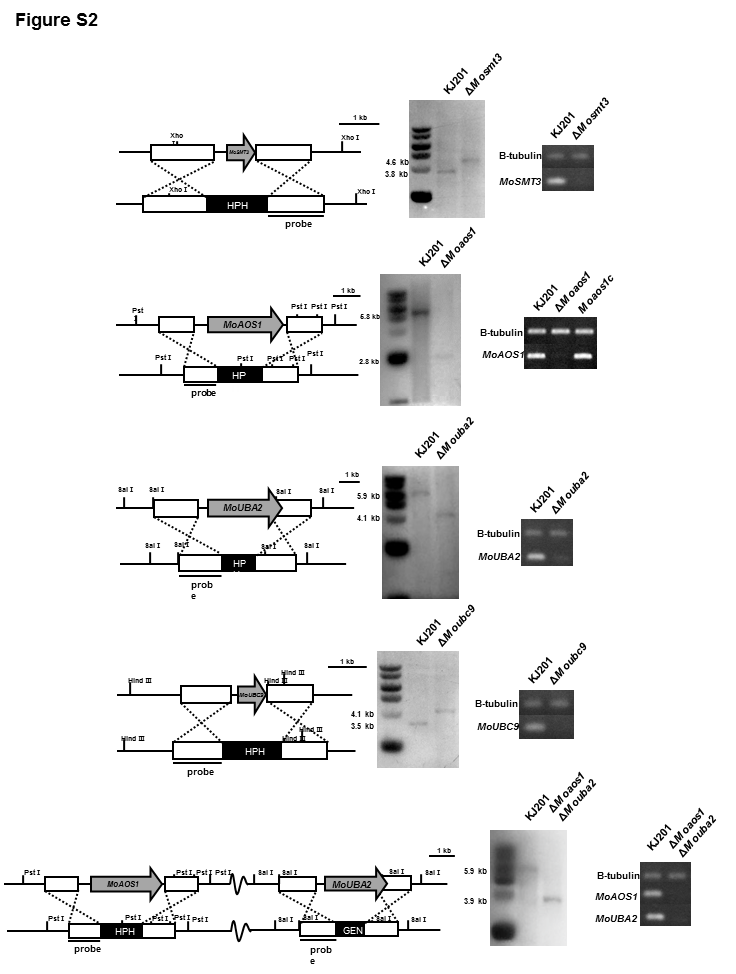

Supplement: Supplementary file 2 — Fig. S2 Southern blot analysis and reverse transcription‐polymerase chain reaction (RT‐PCR) of the deletion mutants. Genomic DNA of wild‐type (WT) and deletion mutants was extracted and digested with PstI, SalI, HindIII or XhoI. The upstream or downstream construct of each gene was used as a probe for Southern blot analysis. Complementary DNA was synthesized from total RNA. β‐tubulin was used for normalization. [file MPP-19-2134-s002.docx]
