# Supplementary material for: SUMOylation is required for fungal development and pathogenicity in the rice blast fungus Magnaporthe oryzae
Source: Mol Plant Pathol. 2018 Jul 17;19(9):2134–48. doi: 10.1111/mpp.12687 (PMC6638150; doi:10.1111/mpp.12687)

**Figure S3. SUMOylation in wild type and** Δ***Moaos1* during conidiation.** Protein extract from the wild type during conidiation was separated on SDS-PAGE and subjected to Western analysis using an anti-HA antibody.


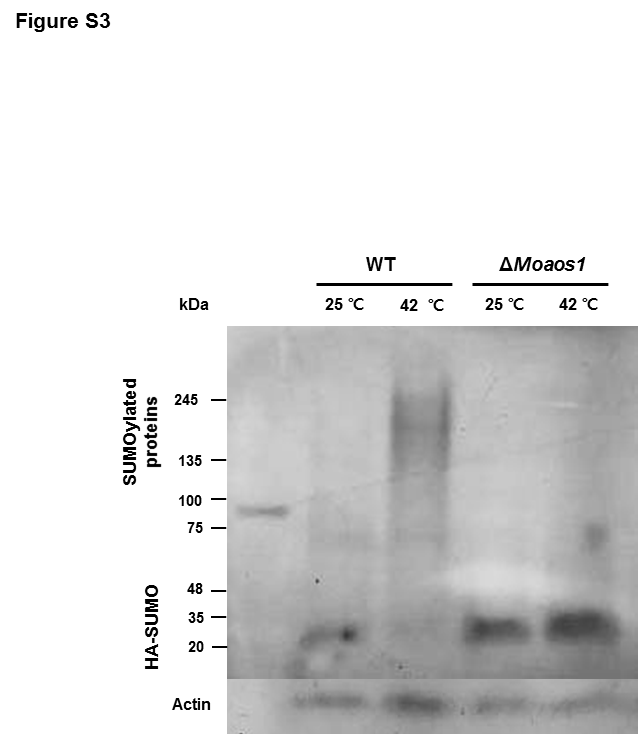

Supplement: Supplementary file 3 — Fig. S3 SUMOylation in wild‐type (WT) and ΔMoaos1 during conidiation. Protein extract from the WT during conidiation was separated by sodium dodecylsulfate‐polyacrylamide gel electrophoresis (SDS‐PAGE) and subjected to Western blot analysis using an anti‐haemagglutinin (HA) antibody. [file MPP-19-2134-s003.docx]
