# Supplementary material for: SUMOylation is required for fungal development and pathogenicity in the rice blast fungus Magnaporthe oryzae
Source: Mol Plant Pathol. 2018 Jul 17;19(9):2134–48. doi: 10.1111/mpp.12687 (PMC6638150; doi:10.1111/mpp.12687)

**Figure S4. SUMOylated proteins in wild type under oxidative stress.** Protein extract from the wild type treated with oxidative stress (100 mM H_2_O_2_) was separated on SDS-PAGE and subjected to Western blot anlaysis using an anti-HA antibody.


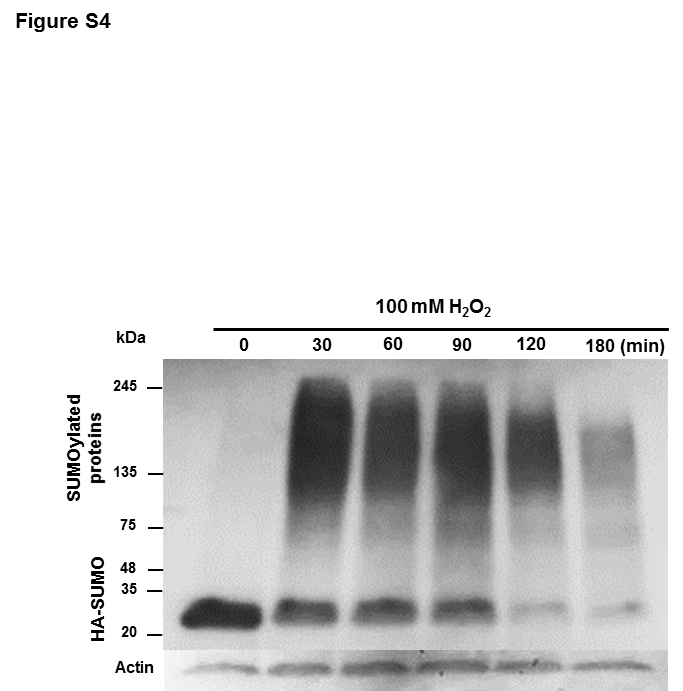

Supplement: Supplementary file 4 — Fig. S4 SUMOylated proteins in wild‐type (WT) under oxidative stress. Protein extract from the WT treated with oxidative stress (100 mm H2O2) was separated by sodium dodecylsulfate‐polyacrylamide gel electrophoresis (SDS‐PAGE) and subjected to Western blot analysis using an anti‐haemagglutinin (HA) antibody. [file MPP-19-2134-s004.docx]
