# Supplementary material for: SUMOylation is required for fungal development and pathogenicity in the rice blast fungus Magnaporthe oryzae
Source: Mol Plant Pathol. 2018 Jul 17;19(9):2134–48. doi: 10.1111/mpp.12687 (PMC6638150; doi:10.1111/mpp.12687)

**Figure S5. Ubiquitination in the wild type,** Δ***Mosmt3,*** Δ***Moaos1*,** Δ***Mouba2* and** Δ***Moubc9*.** Protein extracts of wild type, Δ*Mosmt3,* Δ*Moaos1*, Δ*Mouba2* and Δ*Moubc9* were separated on SDS-PAGE, and subjected to Western blot analysis using an anti-ubiquitin (P4D1) antibody. Ubiquitination was not affected in all of the deletion mutants.


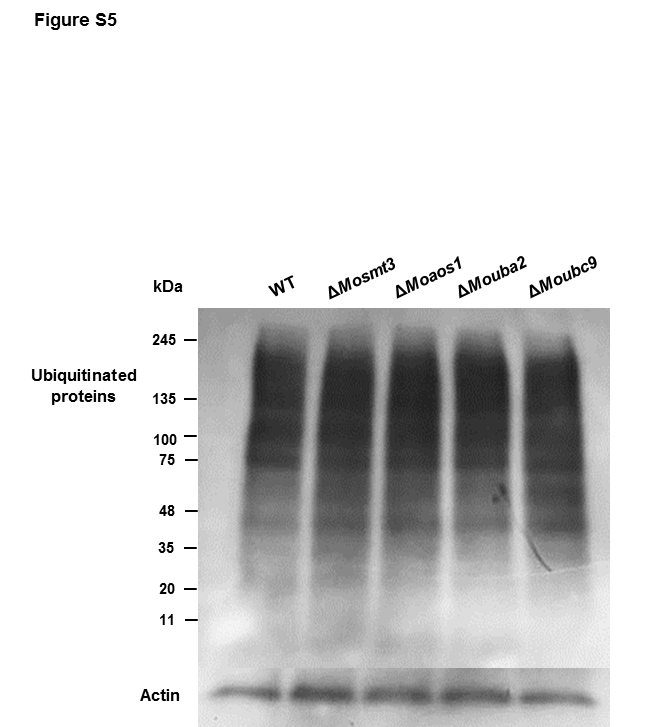

Supplement: Supplementary file 5 — Fig. S5 Ubiquitination in the wild‐type (WT), ΔMosmt3, ΔMoaos1, ΔMouba2 and ΔMoubc9. Protein extracts of WT, ΔMosmt3, ΔMoaos1, ΔMouba2 and ΔMoubc9 were separated by sodium dodecylsulfate‐polyacrylamide gel electrophoresis (SDS‐PAGE) and subjected to Western blot analysis using an anti‐ubiquitin (P4D1) antibody. Ubiquitination was not affected in all of the deletion mutants. [file MPP-19-2134-s005.docx]
