# Supplementary material for: SUMOylation is required for fungal development and pathogenicity in the rice blast fungus Magnaporthe oryzae
Source: Mol Plant Pathol. 2018 Jul 17;19(9):2134–48. doi: 10.1111/mpp.12687 (PMC6638150; doi:10.1111/mpp.12687)

**Figure S6. Defective conidiogenesis was recovered in complemented strains.** Conidiogenesis on conidiophores was observed under a microscope. Scale bar, 100 µm.


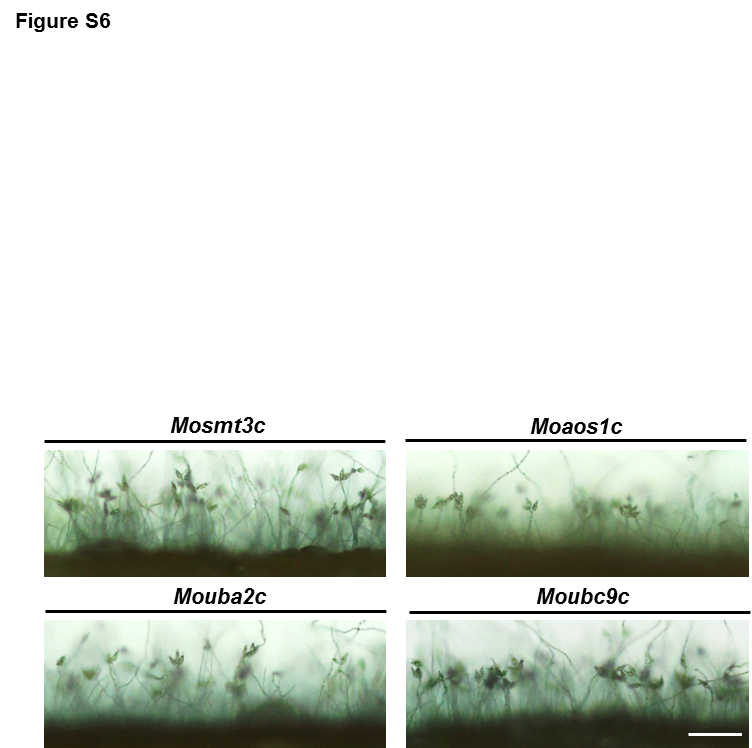

Supplement: Supplementary file 6 — Fig. S6 Defective conidiogenesis was recovered in complemented strains. Conidiogenesis on conidiophores was observed under a microscope. Scale bar, 100 µm. [file MPP-19-2134-s006.docx]
