# Supplementary material for: SUMOylation is required for fungal development and pathogenicity in the rice blast fungus Magnaporthe oryzae
Source: Mol Plant Pathol. 2018 Jul 17;19(9):2134–48. doi: 10.1111/mpp.12687 (PMC6638150; doi:10.1111/mpp.12687)

**Figure S7. Mycelial growth under stress conditions.** Strains were inoculated on nutrient starvation stress (MMA), DNA damage stress (10 mM hydroxyurea and 0.05% methyl methanesulfonate), and oxidative stress (5 mM H_2_O_2_ and 3 mM methyl viologen). Mycelial growth was measured 9 d after inoculation.


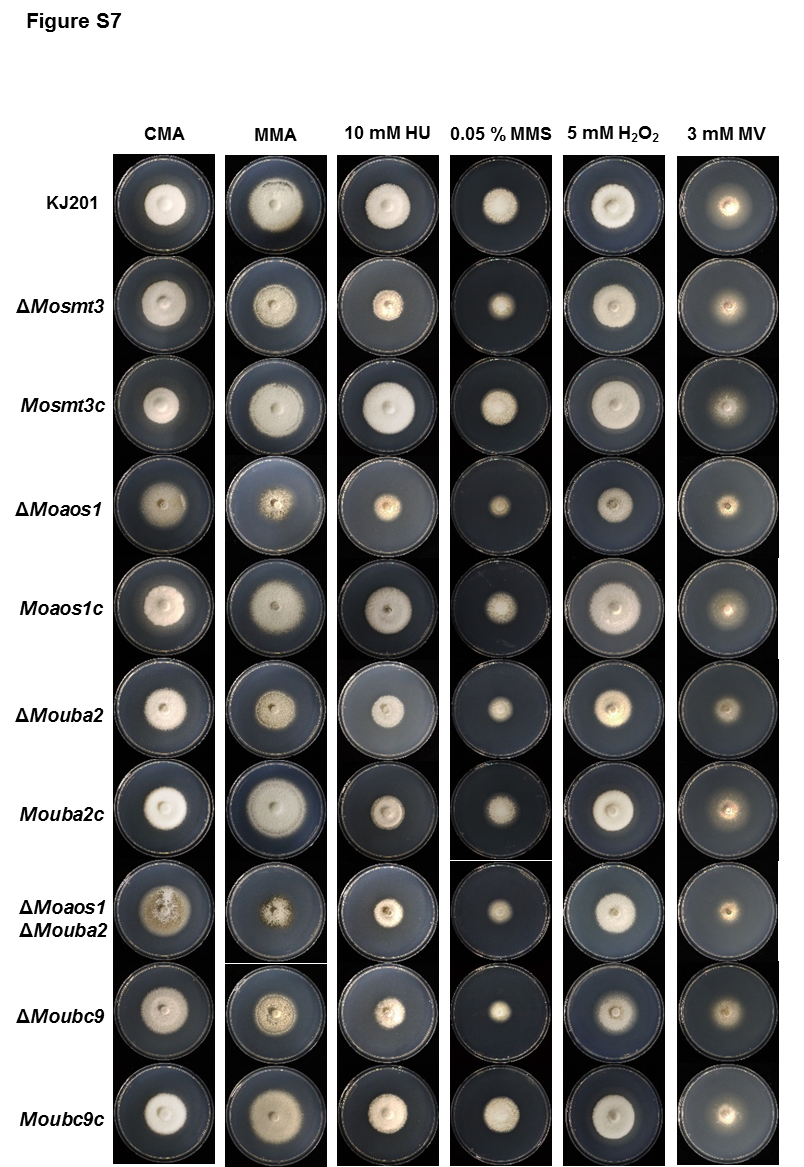

Supplement: Supplementary file 7 — Fig. S7 Mycelial growth under stress conditions. Strains were inoculated on nutrient starvation stress [minimal agar medium (MMA)], DNA damage stress [10 mm hydroxyurea (HU) and 0.05% methyl methanesulfonate (MMS)] and oxidative stress [5 mm H2O2 and 3 mm methyl viologen (MV)]. Mycelial growth was measured 9 days after inoculation. CMA, complete agar medium. [file MPP-19-2134-s007.docx]
