# Supplementary material for: SUMOylation is required for fungal development and pathogenicity in the rice blast fungus Magnaporthe oryzae
Source: Mol Plant Pathol. 2018 Jul 17;19(9):2134–48. doi: 10.1111/mpp.12687 (PMC6638150; doi:10.1111/mpp.12687)

**Figure S8. Invasive hyphae (IH) growth in the complemented strains.** (A) Conidial suspensions (2 × 10^4^/mL) of the complemented strains were inoculated onto 6-week-old rice sheath cells. IH growth was observed under a microscope at 48 hpi. Scale bar, 50 μm. (B) Growth of IH into neighboring cells was classified into three types: Type I for IH restricted to the primary infection cell; Type II for IH growth to adjacent cells; Type III for extensive growth of IH to over adjacent cells.


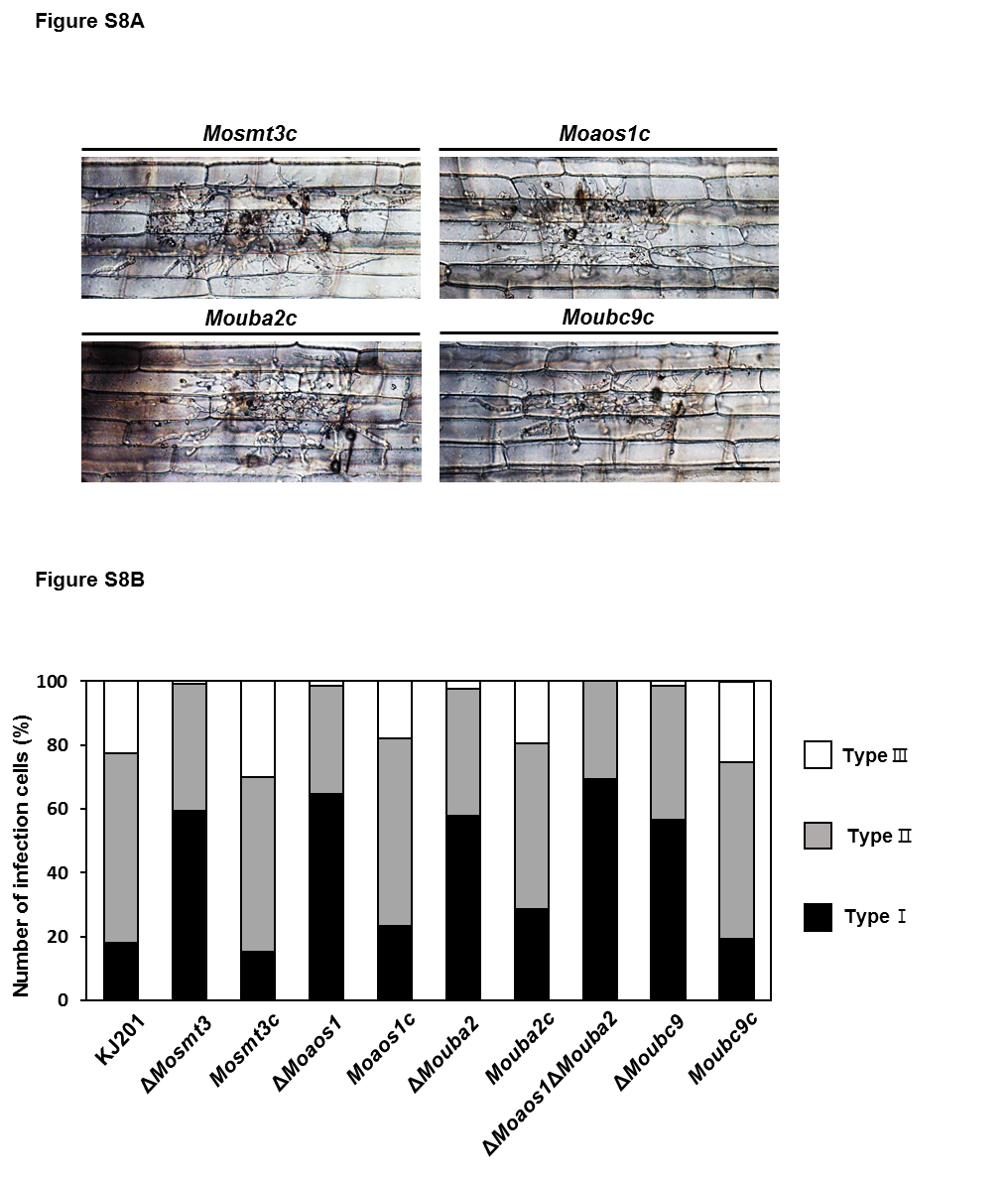

Supplement: Supplementary file 8 — Fig. S8 Growth of invasive hyphae (IH) in the complemented strains. (A) Conidial suspensions (2 × 10^4^/mL) of the complemented strains were inoculated onto 6‐week‐old rice sheath cells. IH growth was observed under a microscope at 48 h post‐inoculation (hpi). Scale bar, 50 μm. (B) Growth of IH into neighbouring cells was classified into three types: Type I for IH restricted to the primary infection cell; Type II for IH growth to adjacent cells; Type III for extensive growth of IH over adjacent cells. [file MPP-19-2134-s008.docx]
