# Supplementary material for: SUMOylation is required for fungal development and pathogenicity in the rice blast fungus Magnaporthe oryzae
Source: Mol Plant Pathol. 2018 Jul 17;19(9):2134–48. doi: 10.1111/mpp.12687 (PMC6638150; doi:10.1111/mpp.12687)

**Figure S9. Penetration assay of the complemented strains.** Conidial suspensions (2 × 10^4^/mL) of the complemented strains were inoculated onto 6-week-old rice sheath cells. Penetration by appressoria at 24 hpi was observed under a microscope. Scale bar, 50 μm.


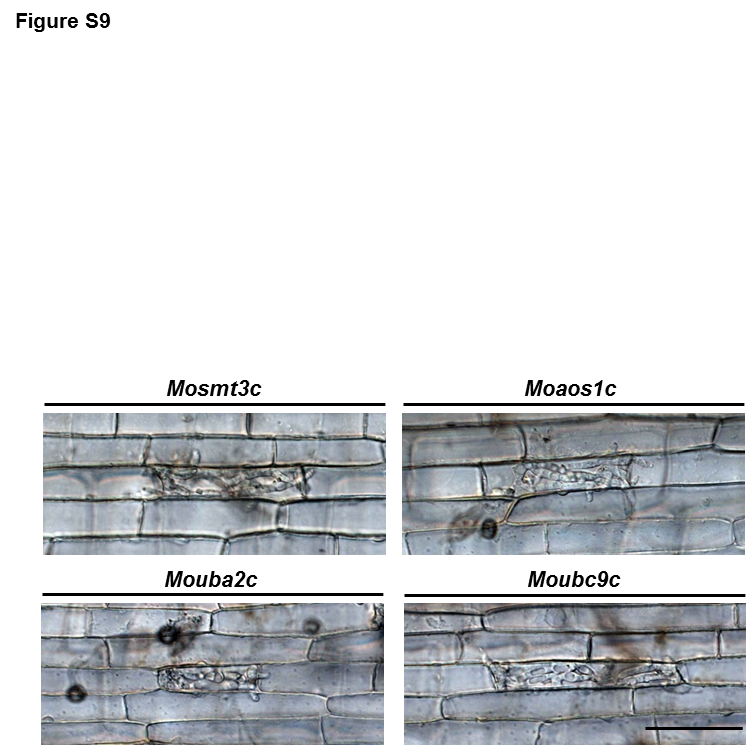

Supplement: Supplementary file 9 — Fig. S9 Penetration assay of the complemented strains. Conidial suspensions (2 × 10^4^/mL) of the complemented strains were inoculated onto 6‐week‐old rice sheath cells. Penetration by appressoria at 24 h post‐inoculation (hpi) was observed under a microscope. Scale bar, 50 μm. [file MPP-19-2134-s009.docx]
