# Supplementary material for: SUMOylation is required for fungal development and pathogenicity in the rice blast fungus Magnaporthe oryzae
Source: Mol Plant Pathol. 2018 Jul 17;19(9):2134–48. doi: 10.1111/mpp.12687 (PMC6638150; doi:10.1111/mpp.12687)

**Figure S10. Intracellular localization of SUMOylation components in *M. oryzae*.** MoAOS1, MoUBA2, MoSMT3 and MoUBC9 were predominantly localized in nuclei during conidial germination and appressorium formation. Scale bar, 50 μm.


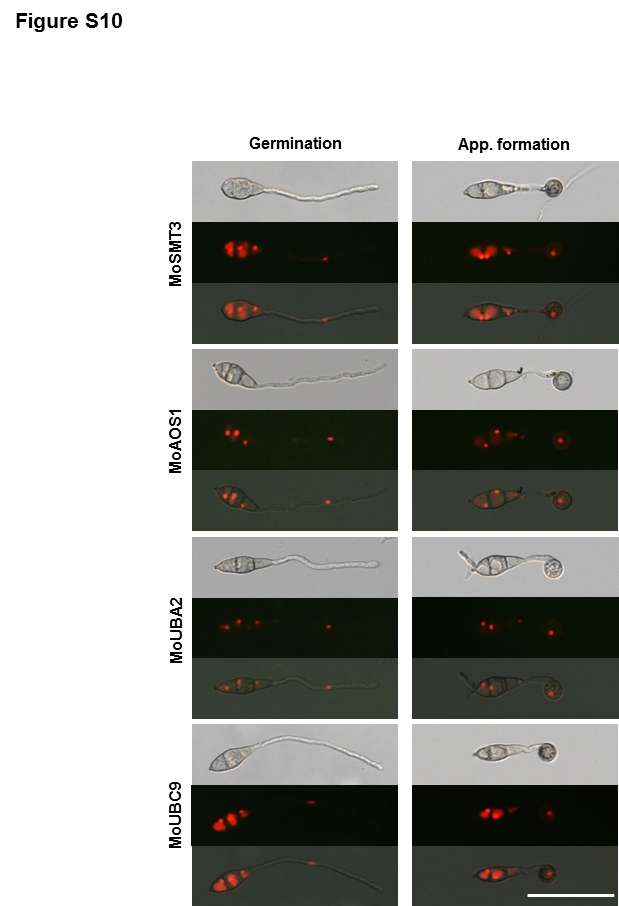

Supplement: Supplementary file 10 — Fig. S10 Intracellular localization of SUMOylation components in Magnaporthe oryzae. MoAOS1, MoUBA2, MoSMT3 and MoUBC9 were predominantly localized in the nuclei during conidial germination and appressorium formation. Scale bar, 50 μm. [file MPP-19-2134-s010.docx]
