# Supplementary material for: SUMOylation is required for fungal development and pathogenicity in the rice blast fungus Magnaporthe oryzae
Source: Mol Plant Pathol. 2018 Jul 17;19(9):2134–48. doi: 10.1111/mpp.12687 (PMC6638150; doi:10.1111/mpp.12687)
Supplement: Supplementary file 11 — Table S1 Domains of Saccharomyces cerevisiae SUMOylation components. [file MPP-19-2134-s011.docx]

**Table S1. Domains of *S. cerevisiae* SUMOylation components**

| **Class** | **Protein** | **Length** | **Pfam ID** | **Pfam description** | **Start** | **End** |
| --- | --- | --- | --- | --- | --- | --- |
| SUMO | SMT3 | 101 | PF11976 | Ubiquitin-2 like Rad60 SUMO-like | 24 | 91 |
| E1 | AOS1 | 347 | PF00899 | ThiF family | 16 | 331 |
|  | UBA2 | 636 | PF00899 | ThiF family | 9 | 437 |
|  |  |  | PF10585 | Ubiquitin-activating enzyme  active site | 280 | 363 |
| E2 | UBC9 | 157 | PF00179 | Ubiquitin-conjugating enzyme | 8 | 148 |
| E3 | SIZ1 | 904 | PF02891 | MIZ/SP-RING zinc finger | 357 | 405 |
|  |  |  | PF02037 | SAP domain | 35 | 68 |
|  |  |  | PF14324 | PINIT domain | 175 | 312 |
|  | SIZ2 | 726 | PF02891 | MIZ/SP-RING zinc finger | 334 | 382 |
|  |  |  | PF02037 | SAP domain | 44 | 77 |
|  |  |  | PF14324 | PINIT domain | 155 | 289 |
|  | MMS21 | 267 | PF11789 | Zinc-finger of the MIZ type in Nse subunit | 171 | 227 |
|  | CST9 | 482 | PF14634 | zinc-RING finger domain | 54 | 92 |
| Protease | ULP1 | 621 | PF02902 | Ulp1 protease family, C-terminal catalytic domain | 447 | 613 |
|  | ULP2 | 1034 | PF02902 | Ulp1 protease family, C-terminal catalytic domain | 456 | 674 |
|  | WSS1 | 269 | PF08325 | WLM domain | 27 | 220 |
