# Supplementary material for: SUMOylation is required for fungal development and pathogenicity in the rice blast fungus Magnaporthe oryzae
Source: Mol Plant Pathol. 2018 Jul 17;19(9):2134–48. doi: 10.1111/mpp.12687 (PMC6638150; doi:10.1111/mpp.12687)
Supplement: Supplementary file 12 — Table S2 The number of Saccharomyces cerevisiae homologues of SUMOylation components in the selected species. [file MPP-19-2134-s012.docx]

**Table S2. The number of *S. cerevisiae* homologs of SUMOylation components in the selected species**

| **Kingdom** | **Phylum** | **Species** | **SUMO** | **E1** | | **E2** | **E3** | | | | **Protease** | | |
| --- | --- | --- | --- | --- | --- | --- | --- | --- | --- | --- | --- | --- | --- |
|  |  |  | **SMT3** | **AOS1** | **UBA2** | **UBC9** | **SIZ1** | **SIZ2** | **MMS21** | **CST9** | **ULP1** | **ULP2** | **WSS1** |
| Fungi | Ascomycota | *Fusarium graminearum* | 1 | 2 | 4 | 15 | 0 | 1 | 1 | 0 | 2 | 1 | 3 |
| Fungi | Ascomycota | *Fusarium oxysporum* | 1 | 4 | 3 | 15 | 1 | 0 | 1 | 0 | 2 | 1 | 2 |
| Fungi | Ascomycota | *Colletotrichum graminicola* | 1 | 2 | 4 | 16 | 1 | 1 | 0 | 0 | 2 | 1 | 3 |
| Fungi | Ascomycota | *Magnaporthe oryzae* | 1 | 4 | 2 | 17 | 1 | 1 | 1 | 0 | 1 | 1 | 3 |
| Fungi | Ascomycota | *Podospora anserina* | 1 | 3 | 4 | 16 | 2 | 0 | 1 | 0 | 1 | 1 | 2 |
| Fungi | Ascomycota | *Neurospora crassa* | 1 | 3 | 3 | 17 | 1 | 0 | 1 | 0 | 1 | 1 | 3 |
| Fungi | Ascomycota | *Botrytis cinerea* | 2 | 3 | 3 | 15 | 1 | 1 | 1 | 0 | 1 | 1 | 1 |
| Fungi | Ascomycota | *Blumeria graminis* | 1 | 2 | 4 | 13 | 1 | 1 | 0 | 0 | 1 | 1 | 1 |
| Fungi | Ascomycota | *Mycosphaerella graminicola* | 2 | 4 | 2 | 20 | 1 | 0 | 0 | 0 | 2 | 0 | 2 |
| Fungi | Ascomycota | *Aspergillus fumigatus* | 1 | 2 | 5 | 19 | 0 | 0 | 1 | 0 | 2 | 1 | 2 |
| Fungi | Ascomycota | *Aspergillus nidulans* | 1 | 2 | 4 | 17 | 2 | 0 | 1 | 0 | 1 | 2 | 2 |
| Fungi | Ascomycota | *Coccidioides immitis* | 0 | 3 | 3 | 15 | 1 | 0 | 1 | 0 | 1 | 1 | 1 |
| Fungi | Ascomycota | *Histoplasma capsulatum* | 1 | 3 | 4 | 14 | 1 | 1 | 0 | 0 | 1 | 1 | 2 |
| Fungi | Ascomycota | *Candida albicans* | 1 | 3 | 3 | 12 | 1 | 0 | 1 | 0 | 2 | 1 | 1 |
| Fungi | Ascomycota | *Saccharomyces cerevisiae* | 1 | 2 | 3 | 14 | 1 | 1 | 1 | 1 | 1 | 1 | 1 |
| Fungi | Ascomycota | *Schizosaccharomyces pombe* | 1 | 3 | 3 | 13 | 1 | 0 | 1 | 0 | 2 | 1 | 2 |
| Fungi | Basidiomycota | *Melampsora laricis-populina* | 5 | 2 | 4 | 15 | 1 | 1 | 0 | 0 | 2 | 1 | 0 |
| Fungi | Basidiomycota | *Puccinia graminis* | 1 | 5 | 7 | 13 | 0 | 2 | 0 | 0 | 2 | 1 | 1 |
| Fungi | Basidiomycota | *Ustilago maydis* | 1 | 2 | 4 | 14 | 1 | 0 | 1 | 0 | 1 | 0 | 0 |
| Fungi | Basidiomycota | *Cryptococcus neoformans* | 1 | 3 | 3 | 14 | 1 | 0 | 0 | 0 | 1 | 0 | 1 |
| Fungi | Basidiomycota | *Serpula lacrymans* | 3 | 3 | 3 | 13 | 1 | 0 | 1 | 0 | 1 | 0 | 3 |
| Fungi | Basidiomycota | *Laccaria bicolor* | 2 | 2 | 4 | 15 | 1 | 0 | 0 | 0 | 1 | 1 | 2 |
| Fungi | Basidiomycota | *Heterobasidion annosum* | 2 | 3 | 3 | 17 | 1 | 0 | 1 | 0 | 1 | 1 | 3 |
| Fungi | Basidiomycota | *Phanerochaete chrysosporium* | 1 | 2 | 4 | 15 | 1 | 0 | 0 | 0 | 1 | 1 | 2 |
| Fungi | Microsporidia | *Encephalitozoon cuniculi* | 1 | 2 | 1 | 8 | 0 | 0 | 0 | 0 | 1 | 0 | 0 |
| Fungi | Chytridiomycota | *Batrachochytrium dendrobatidis* | 1 | 2 | 5 | 17 | 1 | 0 | 1 | 0 | 1 | 1 | 2 |
| Fungi | Blastocladiomycota | *Allomyces macrogynus* | 4 | 5 | 9 | 29 | 1 | 3 | 1 | 0 | 6 | 0 | 1 |
| Fungi | Mucoromycota | *Rhizopus oryzae* | 2 | 1 | 3 | 17 | 0 | 1 | 1 | 0 | 1 | 2 | 1 |
| Fungi | Mucoromycota | *Phycomyces blakesleeanus* | 1 | 3 | 3 | 16 | 3 | 0 | 1 | 0 | 2 | 3 | 1 |
| Animal | Chordata | *Mus musculus* | 6 | 7 | 11 | 48 | 2 | 13 | 0 | 0 | 12 | 5 | 0 |
| Animal | Chordata | *Homo sapiens* | 12 | 23 | 16 | 101 | 2 | 14 | 0 | 0 | 10 | 8 | 0 |
| Animal | Arthropoda | *Drosophila melanogaster* | 1 | 5 | 2 | 34 | 0 | 17 | 0 | 0 | 3 | 3 | 0 |
| Animal | Nematoda | *Caenorhabditis elegans* | 1 | 2 | 6 | 19 | 6 | 0 | 0 | 0 | 1 | 1 | 0 |
| Stramenopiles | Oomycota | *Phytophthora infestans* | 1 | 2 | 7 | 14 | 0 | 1 | 1 | 0 | 6 | 1 | 1 |
| Plant | Streptophyta | *Oryza sativa* | 6 | 4 | 12 | 44 | 0 | 3 | 0 | 0 | 2 | 4 | 2 |
| Plant | Streptophyta | *Arabidopsis thaliana* | 13 | 11 | 9 | 68 | 0 | 6 | 0 | 0 | 9 | 2 | 3 |
