# Supplementary material for: SUMOylation is required for fungal development and pathogenicity in the rice blast fungus Magnaporthe oryzae
Source: Mol Plant Pathol. 2018 Jul 17;19(9):2134–48. doi: 10.1111/mpp.12687 (PMC6638150; doi:10.1111/mpp.12687)
Supplement: Supplementary file 13 — Table S3 The closest Saccharomyces cerevisiae homologues of SUMOylation components in Magnaporthe oryzae. [file MPP-19-2134-s013.docx]

**Table S3. The closest *S. cerevisiae* homologs of SUMOylation components in *M. oryzae***

| **Class** | ***S. cerevisiae* protein** | ***M. oryzae* accession** | **Seq. similarity (%)** |
| --- | --- | --- | --- |
| SUMO | SMT3 | MGG_05737 | 77 |
| E1 | AOS1 | MGG_01669 | 60 |
|  |  | MGG_01832 | 48 |
|  |  | MGG_01409 | 37 |
|  |  | MGG_05569 | 51 |
|  | UBA2 | MGG_06733 | 58* |
|  |  | MGG_09283 | 61 |
| E2 | UBC9 | MGG_00970 | 81 |
|  |  | MGG_04081 | 55 |
|  |  | MGG_06562 | 54 |
|  |  | MGG_01756 | 52 |
|  |  | MGG_00103 | 50 |
|  |  | MGG_03728 | 49 |
|  |  | MGG_02568 | 62 |
|  |  | MGG_05478 | 50 |
|  |  | MGG_01807 | 49 |
|  |  | MGG_00600 | 49 |
|  |  | MGG_00180 | 54 |
|  |  | MGG_14071 | 46 |
|  |  | MGG_14266 | 52 |
|  |  | MGG_04388 | 49 |
|  |  | MGG_09913 | 47 |
|  |  | MGG_09977 | 47 |
|  |  | MGG_02446 | 49 |
| E3 | SIZ1 | MGG_08837 | 53 |
|  | SIZ2 | MGG_00249 | 42 |
|  | MMS21 | MGG_01042 | 62 |
|  | CST9 | - | - |
| Protease | ULP1 | MGG_03144 | 45 |
|  | ULP2 | MGG_07283 | 52 |
|  | WSS1 | MGG_01341 | 52 |
|  |  | MGG_13172 | 49 |
|  |  | MGG_02424 | 47 |

The closest yeast homologs are highlighted in grey.
* It has larger sequence coverage.
