# Supplementary material for: SUMOylation is required for fungal development and pathogenicity in the rice blast fungus Magnaporthe oryzae
Source: Mol Plant Pathol. 2018 Jul 17;19(9):2134–48. doi: 10.1111/mpp.12687 (PMC6638150; doi:10.1111/mpp.12687)
Supplement: Supplementary file 14 — Table S4 Developmental phenotypes of the wild‐type (WT), deletion mutants and complemented strains. [file MPP-19-2134-s014.docx]

**Table S4. Developmental phenotypes of the wild type, deletion mutants and complemented strains**

|  |  |  | **Conidial size** | | |  | |  | |
| --- | --- | --- | --- | --- | --- | --- | --- | --- | --- |
| **Strain** | **Growth (mm)** | **Conidiation**  **(10^4^/ml)** | | **Length (μm)** | **Width (μm)** | | **Conidial germination (%)** | | **Appressorium formation**  **(%)** |
| KJ201 | 61.5±0.4 | 48.3±3.8 | | 31.1±3.2 | 9.8±1.6 | | 95.3±0.6 | | 92.3±1.2 |
| Δ*Mosmt3* | 45.9±3.3^***^ | 4.2±1.3^***^ | | 26.5±3.7^***^ | 8.9±1.4 | | 70.3±6.8^***^ | | 70.7±0.6^***^ |
| *Mosmt3c*^a^ | 62.4±2.0 | 43.7±4.2 | | 30.5±3.0 | 8.7±1.0 | | 92.3±0.6 | | 90.0±1.0 |
| Δ*Moaos1* | 53.3±1.5^***^ | 8.4±0.5^***^ | | 26.0±4.3^***^ | 9.1±1.6 | | 75.7±3.1^***^ | | 65.7±3.5^***^ |
| *Moaos1c* | 59.2±2.3 | 44.7±10.3 | | 29.2±2.8 | 9.0±1.1 | | 94.3±0.6 | | 90.7±1.5 |
| Δ*Mouba2* | 53.3±0.5^***^ | 7.2±0.2^***^ | | 25.7±5.1^***^ | 9.7±1.6 | | 76.3±1.5^***^ | | 71.3±4.7^***^ |
| *Mouba2c* | 60.2±1.2 | 39.7±5.7 | | 29.1±2.9 | 8.3±1.0 | | 95.3±1.2 | | 93.0±4.4 |
| Δ*Moaos1*Δ*Mouba2* | 55.2±0.3^***^ | 8.2±0.9^***^ | | 25.3±4.3^***^ | 8.3±1.1 | | 69.3±2.5^***^ | | 66.7±2.1^***^ |
| Δ*Moubc9* | 51.5±1.0^***^ | 4.3±0.3^***^ | | 26.1±4.6^***^ | 8.9±1.2 | | 69.0±3.6^***^ | | 72.0±1.7^***^ |
| *Moubc9c* | 58.6±1.5 | 49.3±7.0 | | 29.7±3.0 | 9.1±1.0 | | 94.7±1.5 | | 92.0±1.0 |

The significance was statistically determined by *t*-test with ****p* < 0.001.

^a^Complemented strains were named after the gene name with c
