# Supplementary material for: SUMOylation is required for fungal development and pathogenicity in the rice blast fungus Magnaporthe oryzae
Source: Mol Plant Pathol. 2018 Jul 17;19(9):2134–48. doi: 10.1111/mpp.12687 (PMC6638150; doi:10.1111/mpp.12687)
Supplement: Supplementary file 15 — Table S5 Conidial germination and appressorium formation of the wild‐type (WT), deletion mutants and complemented strains after incubation for 2, 8 and 24 h. [file MPP-19-2134-s015.docx]

**Table S5. Conidial germination and appressorium formation of the wild type, deletion mutants and complemented strains after incubation for 2 h, 8 h and 24 h**

|  | **Germination** | | |  | **Appressorium**  **formation** | |
| --- | --- | --- | --- | --- | --- | --- |
| **Strain** | **2 h**  **(%)** | **8 h**  **(%)** | **24 h**  **(%)** |  | **8 h**  **(%)** | **24 h**  **(%)** |
| KJ201 | 95.3±0.6 | 95.7±2.5 | 96.3±1.2 |  | 92.3±1.2 | 94.3±0.6 |
| Δ*Mosmt3* | 70.3±6.8^***^ | 80.7±4.0^**^ | 89.3±2.1^**^ |  | 70.7±0.6^***^ | 77.3±3.1^**^ |
| *Mosmt3c*^a^ | 92.3±0.6 | 93.0±0.0 | 94.0±2.6 |  | 90.0±1.0 | 93.7±2.1 |
| Δ*Moaos1* | 75.7±3.1^***^ | 81.0±4.6^**^ | 85.7±3.5^**^ |  | 65.7±3.5^***^ | 72.7±2.5^***^ |
| *Moaos1c* | 94.3±0.6 | 95.7±2.1 | 96.0±1.0 |  | 90.7±1.5 | 93.0±1.0 |
| Δ*Mouba2* | 76.3±1.5^***^ | 87.3±8.7^**^ | 88.0±1.0^**^ |  | 71.3±4.7^***^ | 76.7±2.5^***^ |
| *Mouba2c* | 95.3±1.2 | 96.7±1.5 | 97.0±1.0 |  | 93.0±4.4 | 93.7±1.2 |
| Δ*Moaos1*Δ*Mouba2* | 69.3±2.5^***^ | 81.3±2.1^**^ | 84.0±2.6^**^ |  | 66.7±2.1^***^ | 70.0±1.7^***^ |
| Δ*Moubc9* | 69.0±3.6^***^ | 85.0±2.0^**^ | 87.7±2.3^**^ |  | 72.0±1.7^***^ | 73.7±4.2^***^ |
| *Moubc9c* | 94.7±1.5 | 95.7±1.2 | 96.0±1.0 |  | 92.0±1.0 | 93.0±1.7 |

The significance was statistically determined by *t*-test with ***p* < 0.05 and ****p* < 0.001.

^a^Complemented strains were named after the gene name with c
