# Supplementary material for: SUMOylation is required for fungal development and pathogenicity in the rice blast fungus Magnaporthe oryzae
Source: Mol Plant Pathol. 2018 Jul 17;19(9):2134–48. doi: 10.1111/mpp.12687 (PMC6638150; doi:10.1111/mpp.12687)
Supplement: Supplementary file 16 — Table S6 List of the strains used in this study. [file MPP-19-2134-s016.docx]

**Table S6. List of strains used in this study**

| **Strain** | **Description** | **Source** |
| --- | --- | --- |
| KJ201 | *M. oryzae*, wildtype strain | CFGR |
| Δ*Mosmt3* | *M. oryzae*, *SMT3* deletion mutant | This study |
| *Mosmt3*c | *M. oryzae*, genetic complementation strain of the above *SMT3* deletion mutant | This study |
| Δ*Moaos1* | *M. oryzae*, *AOS1* deletion mutant | This study |
| *Moaos1c* | *M. oryzae*, genetic complementation strain of the above *AOS1* deletion mutant | This study |
| Δ*Mouba2* | *M. oryzae*, *UBA2* deletion mutant | This study |
| *Mouba2*c | *M. oryzae*, genetic complementation strain of the above *UBA2* deletion mutant | This study |
| Δ*Moaos1*Δ*Mouba2* | *M. oryzae,* *UBA2* deletion mutant of the above *AOS1* deletion mutant | This study |
| Δ*Moubc9* | *M. oryzae*, *UBC9* deletion mutant | This study |
| *Moubc9*c | *M. oryzae*, genetic complementation strain of the above *UBC9* deletion mutant | This study |
| Mav203 | Two hybrid yeast strain (MATα, *leu*2-3,112, *trp*1-901, *his*3Δ200, *ade*2-101, *gal*4Δ, *gal*80Δ, *SPAL*10*::URA*3, *GAL*1::*lac*Z, *HIS*3UAS GAL1*::HIS*3@*LYS*2, *can*1R, *cyh*2R) | Invitrogen |
