# Supplementary material for: SUMOylation is required for fungal development and pathogenicity in the rice blast fungus Magnaporthe oryzae
Source: Mol Plant Pathol. 2018 Jul 17;19(9):2134–48. doi: 10.1111/mpp.12687 (PMC6638150; doi:10.1111/mpp.12687)
Supplement: Supplementary file 17 — Table S7 Primer sequences used in this study. [file MPP-19-2134-s017.docx]

**Table S7. Primer sequence used in this study**

| **Name** | **Sequence 5’→3’** |
| --- | --- |
| MGG_01669_5'flnk_F | GGTTACCCTCTAGAGAGTCCTG |
| MGG_01669_5'flnk_R | CCTCCACTAGCTCCAGCCAAGCCCGTTGGTGTAGTCTCTCTACC |
| MGG_01669_3'flnk_F | GTTGGTGTCGATGTCAGCTCCGGAGTTCGTCCCGGTATGGGTATTG |
| MGG_01669_3'flnk_R | CCAACCAGGGTAGGATAAGGG |
| MGG_01669_nested_F | GGGTTCAGGTCCAGTATAGAGC |
| MGG_01669_nested_R | ACTTAGGTACCACGGACAGC |
| MGG_01669_qRT_F | CAGGCTCCCATTCAAAACACC |
| MGG_01669_qRT_R | CGTTGCTGCTATCCACAGC |
| MGG_06733_5'flnk_F | GGAGAAGAAAGTCGACCCAGTG |
| MGG_06733_5'flnk_R | CCTCCACTAGCTCCAGCCAAGCCGACAGCCCTAACGGACTGAAG |
| MGG_06733_3'flnk_F | GTTGGTGTCGATGTCAGCTCCGGAGCACCTCTTATCGTACAGCTCTCG |
| MGG_06733_3'flnk_R | GAATGCTCACCTCAACGCTGCT |
| MGG_06733_nested_F | GCGTTTGGGACTGAACCCT |
| MGG_06733_nested_R | GAGCATAAGATACCTGCGGCAA |
| MGG_06733_qRT_F | GATTTATCACGCGCCACTCTCG |
| MGG_06733_qRT_R | GTTCGGTCAGCAGTTTTGGCAAG |
| MGG_00970_5'flnk_F | CTGGAGCTGCTTGGCAAATTCATC |
| MGG_00970_5'flnk_R | CCTCCACTAGCTCCAGCCAAGCCGCTTACGGACTGGATGGTC |
| MGG_00970_3'flnk_F | GTTGGTGTCGATGTCAGCTCCGGAGCCAGAAATCGAGTTGGCTCTC |
| MGG_00970_3'flnk_R | GTTGGGGTAGATCCAATTGGTGATGG |
| MGG_00970_nested_F | GAGCTTTACTGCCGAGAACTC |
| MGG_00970_nested_R | GAACAGCGCACAGGCTTTCTC |
| MGG_00970_qRT_F | CTGTTCCATCCCAACGTCTACC |
| MGG_00970_qRT_R | GGTTGTATGCGTCTGCCTGAG |
| MGG_05737_5'flnk_F | CCTCATCAATGTCATCGGCCTTC |
| MGG_05737_5'flnk_R | CCTCCACTAGCTCCAGCCAAGCCCAGAGGCGCAAAGTGAAAGGC |
| MGG_05737_3'flnk_F | GTTGGTGTCGATGTCAGCTCCGGAGCGAGACATTCAGTCAGTTTGGG |
| MGG_05737_3'flnk_R | GCTGAAGTTTGAGAGGGTTGG |
| MGG_05737_nested_F | AGAGTCGCGGAGCAAGACTATTC |
| MGG_05737_nested_R | CGTGTGTTGTTTGTGCCAATCTCG |
| MGG_05737_qRT_F | GGTGACGGACAACAATAACGAG |
| MGG_05737_qRT_R | GGTGACGGACAACAATAACGAG |
| Hyg_F | GGCTTGGCTGGAGCTAGTGGAGG |
| Hyg_R | CTCCGGAGCTGACATCGACACCAAC |
| 01669 Y2H_F_attB | AAAAAGCAGGCTTAATGCAGGACTCAATGACAGC |
| 01669 Y2H_R_attB | AGAAAGCTGGGTACAAGCCCATATAATTACTGTCC |
| 06733 Y2H_F_attB | AAAAAGCAGGCTTAATGGCGACAAATCCCGAGTC |
| 06733 Y2H_R_attB | AGAAAGCTGGGTACTCTCAATCCTCGTCGTCTG |
| 05737 promoter_F_EcoRⅠ | GAATTCCAACTAGCCTTGATGACG |
| 05737 promoter_R_Sal I | GTCGACTATGGAAGTGGATTGTCTCG |
| 05737 _F_HA_Sal I | GTCGACATGTACCCATACGATGTTCCAGATTACGCTATGTCGGATCGCGA |
| 05737_R_Apa I | GGGCCCTTAAGCACCACCAACCTG |
| 01669_EF1α_F | CCCAATCTTCAAAATGCAGGACTCAATGACAGC |
| 01669_attb_R | AGAAAGCTGGGTACTGTCCAAGGGTCATGTC |
| 06733_EF1α_F | CCCAATCTTCAAAATGGCGACAAATCCCGAGTC |
| 06733_attb_R | AGAAAGCTGGGTAATCCTCGTCGTCTGAAATGAC |
| 00970_EF1α_F | CCCAATCTTCAAAATGTCTCTCAGCCAGAACCG |
| 00970_attb_R | AGAAAGCTGGGTAAGGGGCTGGGTTCTCCCT |
| EF1α_Xba I_F | TCTAGACGGTACCTATAGGGCGAA |
| EF1α_R | TTTGAAGATTGGGTTCCTTTTG |
| RFP_EF1α_F | CCCAATCTTCAAAATGGCCTCCTCCGAGGAC |
| RFP_Hind III_R | AAGCTTGGCGCCGGTGGAGT |
| 05737_Hind III_F | AAGCTTATGTCGGATCGCGAGAAC |
| 05737 R | TTAAGCACCACCAACCTGCTC |
| Trpc terminator_05737_F | GGTGGTGCTTAAGATCCACTTAACGTTACTG |
| Trpc terminator_Xho I_R | CTCGAGAAGAAGGATTACCTCTAAAC |
| attb_F | GGGGACAAGTTTGTACAAAAAAGCAGGCT |
| attb_R | GGGGACCACTTTGTACAAGAAAGCTGGGT |
| pDEST22_AD_F | TATAACGCGTTTGGAATCACT |
| pDEST22,32_AD,BD_R | AGCCGACAACCTTGATTGGAGAC |
| pDEST32_BD_F | AACCGAAGTGCGCCAAGTGTCTG |
| COS1_qRT_F | TGCACCACGATCCCAGAGA |
| COS1_qRT_R | GCGATGTTGTGCCGTTGTTCC |
| COM1_qRT_F | GCCAGAGGTCCGCTATCAAA |
| COM1_qRT_R | CGGGATCTCGTCACTGGATT |
| CON7_qRT_F | TAAGGAGATCCGCAAAGAGT |
| CON7_qRT_R | TAGCGTTGTAGTCGGGGAGT |
| HOX2_qRT_F | TGGGGTTCTGCAGCCATGTT |
| HOX2_qRT_R | GTCCCGTGGTGTTACGTTCTGG |
